# BNIP‐2 Activation of Cellular Contractility Inactivates YAP for H9c2 Cardiomyoblast Differentiation

**DOI:** 10.1002/advs.202202834

**Published:** 2022-08-17

**Authors:** Darren Chen Pei Wong, Jingwei Xiao, Ti Weng Chew, Meng Pan, Chang Jie Mick Lee, Jing Wen Ang, Ivan Yow, T. Thivakar, Matthew Ackers‐Johnson, Nicole Jia Wen Lee, Roger Sik‐Yin Foo, Pakorn Kanchanawong, Boon Chuan Low

**Affiliations:** ^1^ Mechanobiology Institute Singapore National University of Singapore Singapore 117411 Singapore; ^2^ Department of Biological Sciences National University of Singapore Singapore 117558 Singapore; ^3^ Genome Institute of Singapore Agency for Science Technology and Research Singapore 138672 Singapore; ^4^ Cardiovascular Research Institute National University Healthcare Systems Singapore 117599 Singapore; ^5^ Department of Medicine Yong Loo Lin School of Medicine National University of Singapore Singapore 117597 Singapore; ^6^ Department of Biomedical Engineering National University of Singapore Singapore 117583 Singapore; ^7^ NUS College National University of Singapore Singapore 138593 Singapore

**Keywords:** BNIP‐2, cardiomyoblast, cellular contractility, LATS1, mechanotransduction, RhoA, Yes‐associated protein (YAP)

## Abstract

Rho GTPases and Hippo kinases are key regulators of cardiomyoblast differentiation. However, how these signaling axes are coordinated spatiotemporally remains unclear. Here, the central and multifaceted roles of the BCH domain containing protein, BNIP‐2, in orchestrating the expression of two key cardiac genes (cardiac troponin T [cTnT] and cardiac myosin light chain [Myl2]) in H9c2 and human embryonic stem cell‐derived cardiomyocytes are delineated. This study shows that BNIP‐2 mRNA and protein expression increase with the onset of cTnT and Myl2 and promote the alignment of H9c2 cardiomyocytes. Mechanistically, BNIP‐2 is required for the inactivation of YAP through YAP phosphorylation and its cytosolic retention. Turbo‐ID proximity labeling corroborated by super‐resolution analyses and biochemical pulldown data reveals a scaffolding role of BNIP‐2 for LATS1 to phosphorylate and inactivate YAP in a process that requires BNIP‐2 activation of cellular contractility. The findings identify BNIP‐2 as a pivotal signaling scaffold that spatiotemporally integrates RhoA/Myosin II and LATS1/YAP mechanotransduction signaling to drive cardiomyoblast differentiation, by switching the genetic programming from YAP‐dependent growth to YAP‐silenced differentiation. These findings offer insights into the importance of scaffolding proteins in bridging the gap between mechanical and biochemical signals in cell growth and differentiation and the prospects in translational applications.

## Graphical abstract

Proper heart formation requires distinct steps of cardiomyocyte proliferation followed by terminal differentiation. This study identifies the scaffold protein, BNIP‐2, as a regulator of this process by performing multifaceted roles in regulating biochemical and mechanotransduction pathways. BNIP‐2 scaffolding of LATS1 and YAP is required to inactivate YAP to induce cardiac genes. Concomitantly, BNIP‐2 regulation of force through RhoA is required for BNIP‐2, LATS1 and LATS1, YAP interaction.

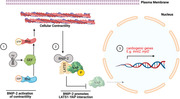

## 1 Introduction

The understanding of differentiation cues that modulate growth, differentiation, and tissue organization of cardiomyocytes is essential for developmental biology and regenerative medicine.^[^ ^1^, ^2^ ^]^ However, in contrast to skeletal muscles, which are readily replenished by residential satellite cells,^[^ ^3^ ^]^ adult cardiomyocytes have limited regenerative potential.^[^ ^1^ ^]^ Hence, it is pertinent to the interest of translational medicine to define mechanistic pathways that integrate biochemical and mechanical signaling. The Rho GTPases and Hippo kinases pathways have emerged as crucial players during cardiomyoblast differentiation.^[^ ^4^, ^5^ ^]^ For example, cardiac troponin is the substrate of the Rho kinase downstream of RhoA GTPases,^[^ ^6^ ^]^ and its regulation is important for cardiac hypertrophy.^[^ ^7^ ^]^ On the other hand, the Hippo pathway can also come under the regulation of Rho GTPases and has been attributed to cardiomyocyte proliferation and heart size regulation.^[^ ^8^ ^]^ To ensure proper regulation of the complicated relationships of these essential signaling axes, we hypothesize that a molecular scaffold is required to orchestrate their signaling activities with spatial and temporal precision. However, any detailed mechanistic understanding of these activities at the biochemical and molecular levels would first be interrogated with relevant cellular models so as to establish further links for in vivo studies.

The H9c2 cardiomyoblast is a valuable in vitro model to delineate mechanical and molecular events that are spatiotemporally coordinated during cardiomyoblast differentiation. H9c2 cells show high similarities to primary cardiomyocytes, including induction of cardiac genes, cytoskeletal rearrangement and hypertrophy response.^[^ ^9^, ^10^ ^]^ Furthermore, H9c2 has been established as an invaluable in vitro model for various cardiac pathophysiological studies including cardiac toxicity testing and ischemia‐reperfusion therapeutic interventions.^[^ ^11^, ^12^, ^13^ ^]^ Hence, this study aims to unveil the spatiotemporal coordination of any molecular events that permit induction of cardiac genes in H9c2 cells, so as to provide novel conceptual insights for future cardiac‐related studies in vitro and in vivo.

The small GTPase RhoA is an upstream regulator of myosin II‐dependent contractility.^[^ ^14^ ^]^ Its activation has been shown to increase actomyosin‐based cellular contractility for mechanotransduction, heart development^[^ ^14^ ^]^ and cardiomyocyte hypertrophy in mature cardiomyocytes.^[^ ^15^ ^]^ However, the modes of action of Rho GTPases in the regulation of cardiac gene expressions are still unclear. RhoA‐mediated generation of actomyosin contractility is a mechanical stimulus that regulates Hippo‐kinase pathway and in turn, influences cell fate by altering subcellular localization of the transcriptional coactivator YAP.^[^ ^16^ ^]^ The canonical Hippo‐kinase pathway consists of MST and LATS, which phosphorylates the transcription coregulators YAP to inactivate and sequester YAP in the cytoplasm.^[^ ^17^, ^18^, ^19^ ^]^ The core kinases, MST and LATS, are sequentially phosphorylated for their activation. However, increasing evidences suggest that cytoskeleton integrity and cellular contractility promotes YAP nuclear localization independent of LATS in nonmuscle cells (e.g., epithelial cells and fibroblasts).^[^ ^20^ ^]^ In the heart, activation of YAP in cardiomyocytes stimulates cardiomyocyte proliferation and is responsible for cardiac regeneration.^[^ ^8^ ^]^ However, heart development and regeneration involve a biphasic phase of proliferation and physiological hypertrophy that is accompanied by increased generation of contractile force.^[^ ^8^, ^21^ ^]^ Such delicate regulation of mechanical and biochemical signals during cardiomyoblast differentiation may spatiotemporally regulate YAP intracellular localization for heart gene expression. In addition, how mechanotransduction through Rho GTPases and the core Hippo kinase cascade are integrated in the context of cardiomyoblast differentiation is still obscure.

BNIP‐2 is a BCH domain‐containing scaffold protein that controls cell morphogenesis, motility, and differentiation.^[^ ^22^, ^23^, ^24^, ^25^ ^]^ The BCH domain is an evolutionarily conserved subset of the CRAL_TRIO/Sec14p superfamily.^[^ ^26^ ^]^ BCH domain‐containing proteins can form homodimers and heterodimers with other BCH domain‐containing proteins and have been demonstrated as a versatile scaffold that regulates small GTPases MAPK and metabolic signaling^[^ ^23^ ^]^ and that it engages Rho via a novel Rho‐binding and lipid‐binding motif.^[^ ^27^ ^]^ Recently, BNIP‐2 has been reported to be a scaffold protein which engage GEF‐H1 to regulate RhoA activation and cell migration in cancer cells.^[^ ^28^ ^]^ In addition, BNIP‐2 is important for skeletal muscle differentiation by CDO‐dependent p38MAPK activation.^[^ ^24^, ^29^ ^]^ However, whether and how BNIP‐2 participates as a scaffold protein during cardiomyoblast differentiation is unknown. As YAP is a mechanosensitive transcriptional coactivator, we hypothesize that BNIP‐2 regulates RhoA activity and actomyosin‐based contractility upstream of Hippo‐kinase pathway to regulate cardiac gene expression.

In this study, we delineated the central and multifaceted roles of BNIP‐2 in integrating mechanical and biochemical signaling for cardiomyoblast differentiation. We showed that BNIP‐2 mRNA and protein expression are upregulated and precedes the fetal heart gene expression in both models of H9c2 cardiomyoblast and human embryonic stem cell derived cardiomyocyte (hESC‐CM). BNIP‐2 depletion severely delayed this process, which was accompanied by reduced contractile force and the activation of several key target genes of YAP (e.g., *CTGF* and *Myc*). With a combination of biochemical analyses corroborated by biophysical and super‐resolution analyses, we show that BNIP‐2 activation of RhoA was responsible for the increased actomyosin‐based intracellular contractility and extracellular traction force. The activation of intracellular force subsequently led to BNIP‐2 scaffolding of LATS1 with YAP in the cytoplasm. Such scaffolding effect directly induced LATS1 to phosphorylate YAP, preventing its nuclear entry, thereby switching the genetic programming from YAP‐dependent growth to cardiomyoblast differentiation. Our findings therefore reveal a pivotal role of BNIP‐2 as a signaling scaffold that spatially and temporally integrates RhoA/Myosin II (mechanical signaling) and MST/LATS/YAP (biochemical signaling) as key mechanotransduction axes to enable cardiac gene expression and cardiomyoblast differentiation.

## 2 Results

### 2.1 Induction of Cardiac Gene Expressions in H9c2 Cardiomyoblast Correlates with Increased BNIP‐2 Expression

To investigate the importance of BNIP‐2 for heart development, we first examined its expression profile during fetal heart development and adult heart by analyzing publicly available databases.^[^ ^30^, ^31^ ^]^ As shown in Figure S1A,B (Supporting Information), BNIP‐2 showed relatively higher expression in cells of mesoderm lineage (e.g., adipose, cardiac, and skeletal muscles), with the ventricular cardiomyocytes showing slightly higher expression compared to atrial cardiomyocytes. Furthermore, BNIP‐2 expression increased during human fetal heart development and was higher compared to other tissues at week 20 (Figure S1C, Supporting Information).^[^ ^31^, ^32^ ^]^ Since BNIP‐2 showed higher expression in ventricular than atrial cardiomyocytes, we proceeded to using the neonate rat ventricular cardiomyoblast (H9c2) that responds to all‐*trans*‐retinoic acid (ATRA)‐induced differentiation as a model^[^ ^9^, ^10^ ^]^ to understand whether and how BNIP‐2 could affect the induction of cardiac genes (e.g., cTnT and Myl2).

**Figure 1:**
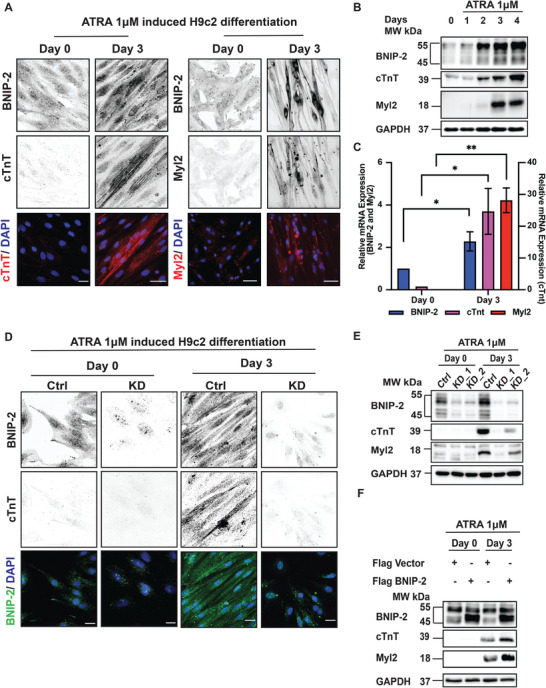
Induction of cardiac gene expressions in H9c2 cardiomyoblast correlates with increased BNIP‐2 expression. A) H9c2 cells were treated with ATRA for 3 d to induce the expression of cardiac markers cTnT and Myl2. Immunofluorescence staining showed that BNIP‐2 expression increased in H9c2 cells expressing cTnT and Myl2. Scale bar = 30 µm, *n* = 3. B) Immunoblot shows that ATRA treatment in H9c2 cardiomyoblast resulted in increased BNIP‐2 expression, concomitant with increased cTnT and Myl2 expressions, *n* = 4. C) RT‐PCR of total mRNA shows a significant increase in BNIP‐2, cTnT, and Myl2 mRNA expressions after 3 d of ATRA treatment. The graph represents mean ± SEM, *n* = 3. *p*‐values were calculated by the Student's *t*‐test: *p < 0.05 and **p < 0.01. D,E) BNIP‐2 knockdown in H9c2 cells reduced the expression of cardiac genes (cTnT and Myl2) after 3 d of ATRA treatment as represented in immunofluorescence and immunoblot, respectively, scale bar = 30 µm and *n* = 4 for both (D) and (E). F) H9c2 cells were treated with ATRA for 3 d. Overexpression of Flag BNIP‐2 in H9c2 cells enhanced the expression of cardiac genes (cTnT and Myl2) compared to control cells transfected with Flag vector, *n* = 3.

**Figure 2:**
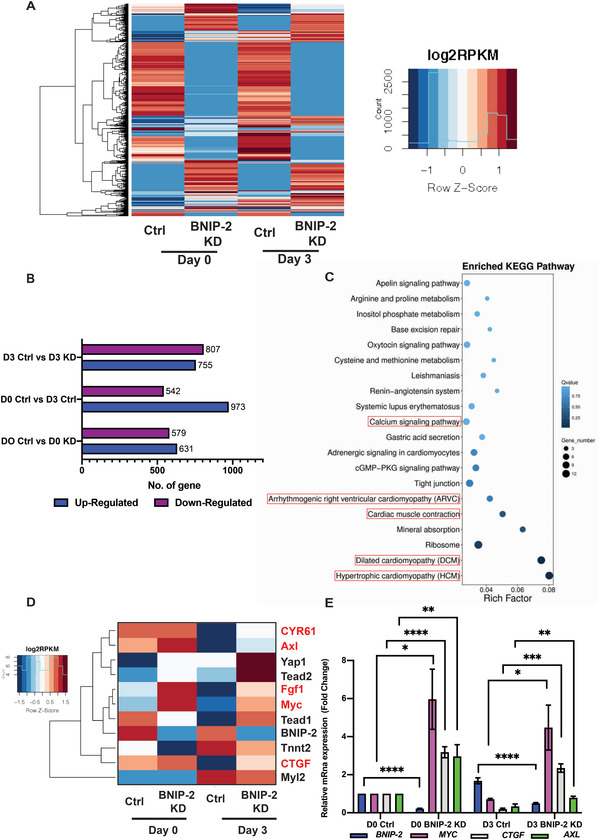
BNIP‐2 knockdown activates YAP target genes in H9c2 cells. A) Heatmap represents genes that were up or downregulated comparing control H9c2 cells to BNIP‐2 knockdown H9c2 cells, and Day 0 versus Day 3 ATRA treated H9c2 cells. Reads per kilobase of transcript, per million mapped reads (RPKM) are indicated by Log2 RPKM. The color indicates the fold change difference (high: red, low: blue). B) The bar graph represents number of genes up or downregulated compared between samples using Poisson distribution algorithms to detect the differential expressed genes. C) KEGG pathway analysis identified pathway differences between Day 3 control versus Day 3 BNIP‐2 knockdown cells. Red boxes highlight pathways related to cardiac signaling. The color in the chart indicates the *q*‐value (high: white, low: blue), lower *q*‐value indicates the more significant enrichment. Point size indicates DEG number (The bigger dots refer to larger amounts). Rich factor refers to the value of enrichment factor, which is the quotient of foreground value (the number of DEGs) and background value (total gene amount). The larger the value, the more significant enrichment. D) BNIP‐2 knockdown increased the expression of YAP regulated transcriptional target genes (highlighted in red) and reduced expression of cardiac markers (cTnT and Myl2). Color intensity (high: red; low: blue) shows fold difference in reads per kilobase of transcript, per million mapped reads (RPKM) of BNIP‐2 knockdown cells against Day 0 and Day 3 control cells. E) RT‐PCR analysis of total mRNA from H9c2 extracts shows that YAP regulated transcriptional targets, MYC, CTGF, and AXL, were increased in BNIP‐2 knockdown cells. Knockdown of BNIP‐2 was also unable to reduce the expressions of MYC, CTGF, and AXL after 3 d of ATRA treatment. The graph represents mean ± SEM, *n* = 5. *p*‐values were calculated by the Student's *t*‐test: *p < 0.05, **p < 0.01, ***p < 0.001, and ****p < 0.0001.

As shown in **Figure** **1**A,B, ATRA treatment induced a significant increase in BNIP‐2 protein expression that paralleled the increased expression of the cardiac genes, cardiac troponin T (cTnT) and cardiac Myosin Light Chain 2 (Myl2), which are also two key components of the force generation machinery in cardiomyocytes.^[^ ^33^ ^]^ Consistently, quantitative real‐time PCR analyses confirmed an increase in BNIP‐2 mRNA (Figure 1C) with the increased expression of cTnT and Myl2. We corroborated these findings by confirming an elevated BNIP‐2 expression in hESC‐CM that preceded the activation of cTnT and Myl2 expression (Figure 2A, Supporting Information).

Next, to determine whether enhanced BNIP‐2 expression was essential for H9c2 cardiomyoblast differentiation, we knocked‐down BNIP‐2 and observed that BNIP‐2 knockdown significantly reduced the expression of cTnT and Myl2 (Figure 1D,E). Conversely, overexpression of BNIP‐2 enhanced the expression of cTnT and Myl2 (Figure 1F). Importantly, overexpression of BNIP‐2 promoted the alignment of dispersed cardiomyocytes that expressed cTnT (Figure 2B, Supporting Information), implying the possibility that H9c2 cells that expressed cTnT and Myl2 could collectively exert extracellular forces.

BNIP‐2 was previously demonstrated to promote C2C12, skeletal muscle cell differentiation by CDO‐dependent p38MAPK activation.^[^ ^24^ ^]^ We next sought to understand if the same pathway would be involved during H9c2 differentiation. Intriguingly, BNIP‐2 knockdown in H9c2 cells had no significant effect on p38MAPK phosphorylation, nor did p38MAPK phosphorylation change over the course of differentiation under ATRA treatment (Figure 2C, Supporting Information). In addition, quantitative RT‐PCR analysis of the surface receptor CDO revealed no significant differences in their gene expression between H9c2 cells expressing high and low levels of cTnT (Figure 2D, Supporting Information).

Collectively, these results strongly suggest that BNIP‐2 promotes cardiac tissue morphogenesis, synchronization of cardiomyocytes alignment and the expression of cardiogenic markers independently of the p38MAPK regulatory pathway. These results prompt us to identify the mechanistic basis by which BNIP‐2 may act as a key regulator to induce cardiac gene expression in H9c2 cells.

### 2.2 RNA Sequencing Revealed Aberrant Cardiac Signaling and Activation of YAP Targets in BNIP‐2 Knockdown H9c2 Cells

We next performed total RNA sequencing of control H9c2 cardiomyoblast compared with BNIP‐2 knockdown H9c2 cells to characterize the signaling pathways affected by BNIP‐2 depletion (**Figure** **2**A–D). Comparing control cells and BNIP‐2 knockdown cells after 3 days of ATRA treatment, we observed significant differences in pathways regulating cardiac signaling (e.g., calcium signaling, cardiac hypertrophy, etc.) (Figure 2C). Interestingly, concomitant with an expected reduced expression of cardiac marker genes (cTnT and Myl2), an upregulation of YAP expression and various YAP target genes were strikingly activated in BNIP‐2 knockdown H9c2 cells (Figure 2D). RT‐PCR analyses further validated an enrichment of expression in several potent YAP target genes such as *ctgf*, *myc*, and *axl* in BNIP‐2 knockdown cells (Figure 2E).

Together, these data confirmed the requirement of BNIP‐2 to induce cardiac gene expression during ATRA‐induced differentiation, possibly by regulating the Hippo‐YAP pathway.

### 2.3 YAP Is Inactivated during Cardiac Gene Induction

**Figure 3:**
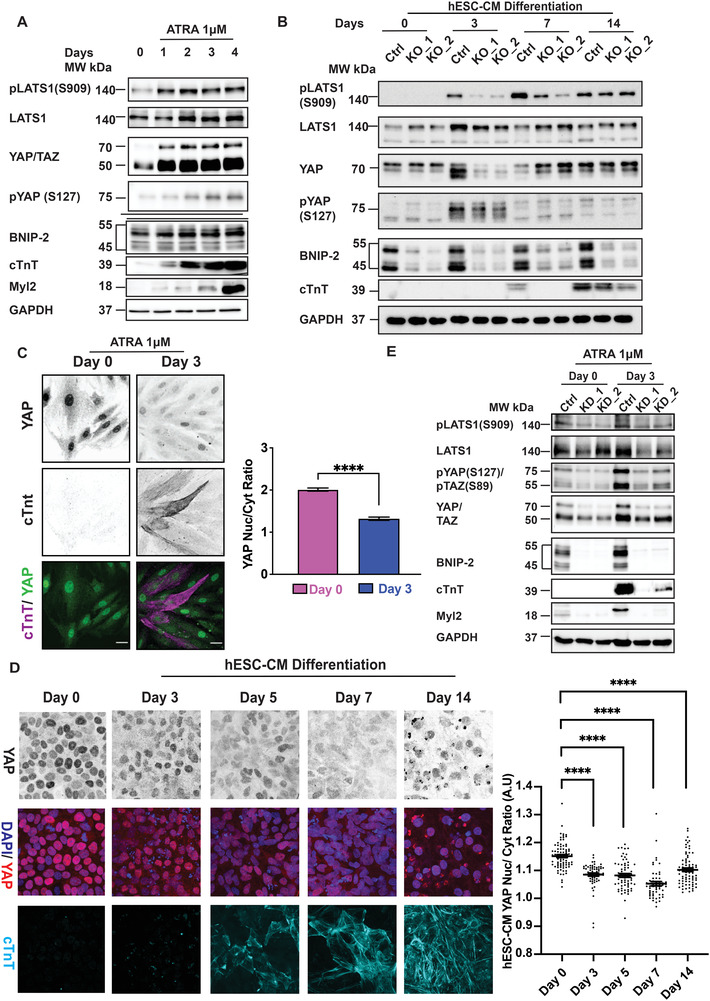
YAP is inactivated during cardiac gene induction. A) Cell lysates from H9c2 cells after ATRA treatment were analyzed for the expression of LATS1, YAP, BNIP‐2, cTnT, and Myl2. Immunoblot showed that LATS1 (pLATS1(S909)) was activated early during differentiation (the induction of cTnT and Myl2), corresponding to an increased in YAP inactivation (pYAP(S127)). This process was also accompanied by increased BNIP‐2 expression (also represented in Figure 1B), *n* = 4. B) Immunoblot shows that BNIP‐2 CRISPR knockout hESC cells had significant delays in the induction of the cardiac marker cTnT. This was also accompanied by reduced LATS1 activation (pLATS1(S909)), YAP inactivation (pYAP(S127)) in hESC‐CM, particularly so on Day 3. *n* = 3. C Immunofluorescence showing H9c2 endogenous YAP preferentially localized to the cytoplasm (lower nuclear/cytoplasmic ratio) after 3 d of ATRA treatment. The graph represents mean ± SEM, *n* = 22. *p*‐value was calculated by the Student's *t*‐test: ****p < 0.0001. D) Immunofluorescence staining and one‐way ANOVA quantification on the right shows preferential YAP nuclear localization during early cardiomyoblast differentiation of hESC‐derived cardiomyocytes. The bars represent mean ± SEM, *n* = 81. *p*‐value was calculated by one‐way ANOVA: ****p < 0.0001. E) Control (Day 0) and 3 d ATRA treated (Day 3) H9c2 cell lysates were harvested for immunoblot analysis. Immunoblot shows BNIP‐2 knockdown impaired H9c2 differentiation (expression of cTnT and Myl2) and LATS1 activation (reduced LATS1(S909)). This was also accompanied by reduced inactivation of YAP (reduced YAP(S127)) in H9c2 cells, *n* = 3.

To test the hypothesis that YAP is inactivated for cardiomyoblast differentiation, we first verified whether LATS1, the direct inactivating kinase of YAP, was activated during cardiomyoblast differentiation. As shown in **Figure** **3**A,B, LATS1 was activated (as shown by its phosphorylation at S909) during differentiation in both H9c2 and hESC‐CM cells. This was also accompanied by increased levels of YAP phosphorylation, which is indicative of its cytoplasmic retention and inactivation. Since phosphorylated YAP accumulates in the cytoplasm,^[^ ^16^, ^34^ ^]^ we next performed immunofluorescence to confirm the subcellular localization of YAP. Interestingly, while YAP in H9c2 and hESC cells preferentially localizes to the cytoplasm in cells expressing cTnT (Figure 3C,D), hESC‐CM showed a biphasic profile for YAP localization, with re‐localization of YAP to the nucleus after 14 d (Figure 3D). This could be partially explained by an increase in hESC‐CM YAP expression from day 7 onward (Figure 3B), which might have overwhelmed LATS1‐dependent inactivation.

Since our results indicate that differentiation and cardiac gene induction by BNIP‐2 activate Hippo pathway (through LATS1 phosphorylation) to inactivate YAP (through YAP phosphorylation at serine 127), we hypothesize that BNIP‐2 directly regulates YAP phosphorylation status and its localization by acting as a scaffold for YAP and LATS1. We set out to verify whether BNIP‐2 knockdown could result in the accumulation of YAP in H9c2 nucleus. As expected, BNIP‐2 knockdown significantly reduced YAP S127 phosphorylation and increased the accumulation of YAP in the nucleus (Figure 3B,E and Figure S3A, Supporting Information), concomitant with reduced LATS1 phosphorylation. Next, a nonphosphorylatable YAP mutant (YAP5SA) that is refractory to LATS regulation and constitutively localized to the nucleus,^[^ ^35^ ^]^ was introduced to H9c2 cells. Indeed, the forced nuclear localization of YAP resulted in reduced expression of cTnT, indicative of reduced differentiation (Figure S3B,C, Supporting Information). To corroborate these observations, we performed a luciferase assay directly measuring YAP activity in control, BNIP‐2 knocked down and BNIP‐2 overexpressed H9c2 and HEK293T cells. As expected, knockdown and overexpression of BNIP‐2 resulted in increased luciferase signal (higher YAP activity) and reduced luciferase signal (lower YAP activity), respectively (Figure S3D, Supporting Information).

Taken together, our results show that: i) BNIP‐2 expression is indispensable for H9c2 and hESC‐CM differentiation (as marked by cTnT and Myl2), and ii) YAP inactivation (through YAP phosphorylation and cytoplasmic localization) is a pre‐requisite for early cardiac gene induction. Importantly, our results strongly suggest that BNIP‐2 serves as a master regulator of YAP inactivation through the activation of LATS1 during heart cell differentiation.

### 2.4 BNIP‐2 Interacts with LATS1 to Promote YAP Cytosolic Localization

**Figure 4:**
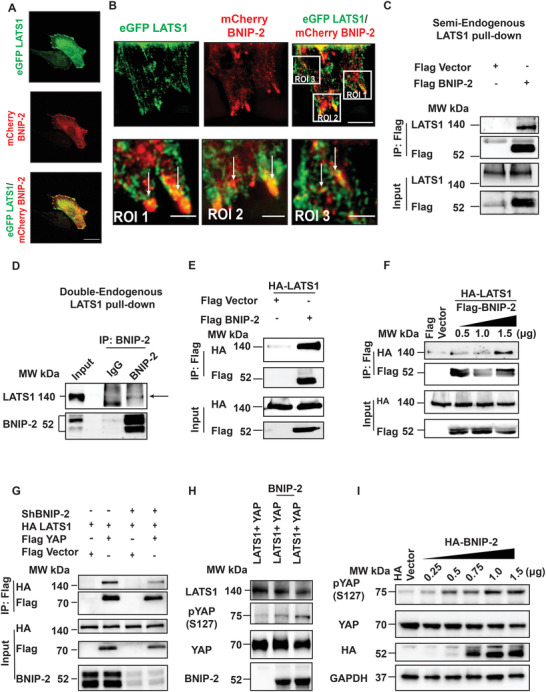
BNIP‐2 interacts with LATS1 to promote YAP cytosolic localization. A) Immunofluorescence shows that endogenous LATS1 (green) and endogenous BNIP‐2 (red) partially colocalized at the cytoplasm and cell edges in H9c2 cells. Scale bar = 30 µm, *n* = 3. B) Super‐resolution using SIM (2.5 µm resolution) microscopy shows eGFP LATS1 (green) and mCherry BNIP‐2 (red) colocalized at cell edges in H9c2 cells. Scale bar = 10 µm and scale bar = 2.5 µm for ROI images (white boxes). C) Immunoblots showing Flag BNIP‐2 co‐immunoprecipitated with endogenous LATS1 (semi‐endogenous pull‐down), *n* = 3. D) Endogenous LATS1 is a pulldown product of endogenous BNIP‐2 in H9c2 cells. Double endogenous pulldown with BNIP‐2 as bait captured LATS1 as an interacting partner, *n* = 3. E) Immunoblot shows that Flag BNIP‐2 co‐immunoprecipitated with HA‐LATS1 in HEK293T cells, suggesting them as interacting partners, *n* = 4. F) Immunoblot shows increased HA LATS1 co‐immunoprecipitated with increasing concentration of Flag BNIP‐2 in HEK293T cells, *n* = 3. G) Immunoblot shows BNIP‐2 knockdown reduced the interaction between Flag YAP and HA LATS1 in HEK293T cells, *n* = 3. H) Immunoblot showing BNIP‐2 promotes LATS1‐induced YAP phosphorylation at serine 127 in an in vitro kinase assay. HA‐BNIP‐2 was translated in vitro and LATS1 and YAP were endogenously immunoprecipitated with protein A/G beads. The reaction was incubated with ATP to assay for YAP phosphorylation, *n* = 3. I) Immunoblot showing increased YAP phosphorylation with increasing concentration of overexpressed BNIP‐2 in H9c2 cells, *n* = 3.

The BCH domains of BNIP‐2 and those present in BPGAP1, BNIP‐H and BNIP‐XL, are known to serve as scaffolds for small GTPases, kinases and metabolic enzymes.^[^ ^22^, ^23^, ^36^ ^]^ This raises the exciting possibility that BNIP‐2 might serve as a scaffold to support the interaction between LATS1 and YAP, thus directly inactivating YAP through its phosphorylation. Notably, we found partial colocalization between BNIP‐2 and LATS1 in the immunofluorescence staining of H9c2 cells (**Figure** **4**A). To further identify the subcellular localization of BNIP‐2 in relation to LATS1, we then performed super‐resolution structured illumination microscopy (SIM). We observed that LATS1 colocalized with BNIP‐2 at the cell protrusions (Figure 4B), which was the intracellular location where BNIP‐2 was previously reported in epithelial cells.^[^ ^37^ ^]^

To examine whether BNIP‐2 could directly interact with any components of the Hippo pathway or other novel regulators of YAP, we performed an unbiased Turbo‐ID proximity labeling assay in culture media containing biotin and using HEK293T cells transfected with 3XHA‐BNIP‐2‐TurboID_pCDNA3 construct as bait.^[^ ^38^ ^]^ Interestingly, LATS1 was identified as a putative binding partner of BNIP‐2 in the mass spectrometry analysis (Figure S4A, Supporting Information), which supports our observation that BNIP‐2 colocalized with LATS1 in immunofluorescence studies (Figure 4A,B). This novel and physiological interaction of LATS with BNIP‐2 was further verified by confirming the presence of endogenous BNIP‐2/LATS complex in H9c2 cells (Figure 4C,D) and by overexpression of Flag‐tagged BNIP‐2 and HA‐tagged LATS1 in HEK293T cells (Figure 4E). Titration experiment with an increasing amount of BNIP‐2 also showed an increased association between LATS1 and BNIP‐2 in a dose‐dependent manner (Figure 4F). Consistently, LATS1 upstream kinase MST1, and LATS1 substrate YAP were identified in the pull‐down of BNIP‐2 in the overexpression system (Figure S4D, Supporting Information).

We next generated a stable BNIP‐2 knockdown HEK293T cell line to functionally determine whether BNIP‐2 affects LATS1 and YAP interaction. Consistent with our hypothesis that BNIP‐2 scaffolds Hippo‐YAP components, knockdown of BNIP‐2 significantly reduced the interaction between LATS1 and YAP (Figure 4G). On the other hand, overexpression of BNIP‐2 enhanced LATS1 and YAP interaction at a dose‐dependent level (Figure S4C, Supporting Information). The drop in LATS1 and YAP interaction at a high dosage of BNIP‐2 is consistent with the classical demonstration of the “scaffolding” effect, namely, an optimal concentration of scaffold promotes interaction. However, too little scaffold is insufficient to bridge, whereas too much scaffold would compete with, the interactions of component proteins (Figure S4D, Supporting Information).^[^ ^17^, ^36^, ^39^ ^]^ Next, we performed an in vitro kinase assay using LATS1, YAP, and BNIP‐2 and showed that BNIP‐2 directly promotes YAP phosphorylation (Figure 4H). Finally, we show that increased BNIP‐2 expression correlates with increased YAP phosphorylation in H9c2 cardiomyoblast (Figure 4I).

Collectively, our data indicate that BNIP‐2 serves as a novel scaffold necessary for the Hippo‐YAP pathway signaling cascade by promoting LATS1‐YAP interaction, which mediates YAP inactivation through YAP phosphorylation and its cytoplasmic retention. As a result, the growth‐promoting transcriptional coactivator role of YAP is switched off (e.g., via myc and ctgf) and turns into differentiation‐enabling mode via the induction of cardiac genes cTnT and Myl2.

### 2.5 BNIP‐2 Activation of Cellular Contractility Inhibits YAP Function

YAP is a mechanosensitive transcription coactivator whose cytoplasmic nuclear shuttling is influenced by intracellular contractility and force exertion from the extracellular matrix.^[^ ^19^, ^20^, ^40^, ^41^ ^]^ While recent studies have shown that mechanical force regulates YAP activity in cellular proliferation and fibroblast spreading by promoting its nuclear localization,^[^ ^18^, ^20^, ^34^ ^]^ little is known about whether this process is recapitulated physiologically in other tissues, especially in the heart. Furthermore, pathophysiologic findings in myocardiac infarction models have shown reduced ventricular contractile force associated with hypertrophy and YAP activation,^[^ ^5^, ^42^ ^]^ suggesting plausible alternative models. Hence, it is pertinent to ascertain whether BNIP‐2 serves a multifaceted role in activating intracellular contractile force to inactivate YAP, instead of activating it.

Our results thus far showed that YAP retention in the cytoplasm is required to drive differentiation (Figure 3), and this mechanism is mediated in part by the ability of BNIP‐2 to scaffold LATS1 and YAP (Figure 4). In addition, the coinduction of BNIP‐2 and contractility proteins (e.g., cTnT and Myl2) (Figure 1), the alignment of H9c2 cells that over expressed BNIP‐2 (Figure S2B, Supporting Information) as well as previously known function of BCH domain as a GTPase regulator, point toward the possible role(s) of intracellular contractile force as a mechanical module regulating cardiomyoblast differentiation governed by BNIP‐2.

**Figure 5:**
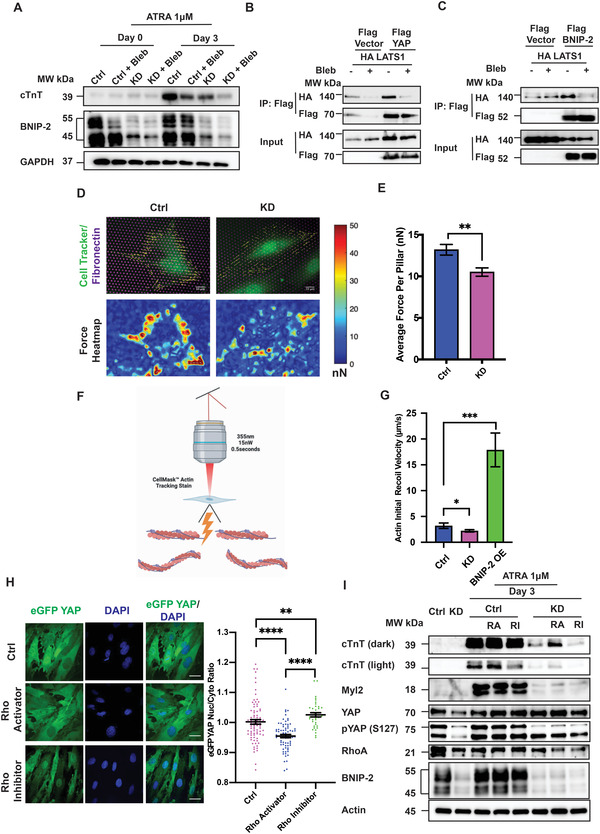
BNIP‐2 activation of cellular contractility promotes YAP cytosolic retention for the induction of cardiac gene. A) Treatment with blebbistatin (20 × 10^−6^ m) or BNIP‐2 knockdown reduced cardiac cTnT expression after 3 d of ATRA treatment in H9c2 cells. Combination of BNIP‐2 knockdown and blebbistatin treatment had a synergistic effect (lower cTnT), *n* = 3. B) Blebbistatin (20 × 10^−6^ m) reduced the interaction between Flag YAP and HA LATS1. Pulldown of HA LATS1 from Flag YAP. C) Blebbistatin (20 × 10^−6^ m) reduced interaction between HA LATS1 and Flag BNIP‐2. Pulldown of HA LATS1 from Flag BNIP‐2. (B) and (C) were repeated three times with similar results. D,E) H9c2 cells were seeded on fibronectin‐coated micropillars. BNIP‐2 knockdown reduced the average force generation on pillar substrate (Scale bar on the right denotes, red: higher force and blue: lower force). Unpaired Student's *t*‐test was used and the bars represent mean ± SEM, *n* = 34. *p*‐value was calculated by Student's *t*‐test: **p < 0.01. F,G) Schematic and quantification showing the initial recoil velocity of actin filaments in H9c2 cells. Knocking down BNIP‐2 reduced the recoil velocity by about 0.3‐folds. Whereas BNIP‐2 overexpression increased the recoil velocity by nearly tenfolds. The graph represents mean ± SEM, *n* = 20. *p*‐values were calculated by Student's *t*‐test: *p < 0.05 and **p < 0.01. H) Rho activation is important for cytoplasmic localization of YAP. Immunofluorescence shows the addition of Rho activator (1 × 10^−6^ m) or inhibitor (1 × 10^−6^ m) influenced YAP cellular localization. Rho activator reduced nuclear YAP while Rho inhibitor increased nuclear YAP. Scale bar = 30 µm. The graph represents mean ± SEM, *n* = 81. *p*‐values were calculated by one‐way ANOVA: **p < 0.01 and ****p < 0.0001. I) Immunoblot showed that Rho activation with Rho activator II (1 µg mL^−1^) partially rescued the induction of cardiac genes (cTnT and Myl2) in BNIP‐2 knockdown cells after 3 d of ATRA treatment. By contrast, Rho inhibition by C3 toxin (1 × 10^−6^ m) further suppressed this process, *n* = 3.

To address this, we first sought to establish whether intracellular contractility is important for differentiation. We found that adding blebbistatin, an inhibitor of nonmuscle and cardiac myosin II, significantly reduced the expression of cTnT in control and more so in BNIP‐2 knockdown cells (**Figure** **5**A). Therefore, a continuous input of cellular contractility appears to be required for differentiation. Since differentiation requires YAP inactivation through BNIP‐2 through the regulation of LATS1 phosphorylation of YAP, we next examined the interaction of LATS1 and YAP, as well as BNIP‐2 and LATS1 in blebbistatin‐treated cells. Interestingly, inhibiting cellular contractility reduced the interactions between LATS1 and YAP, and between BNIP‐2 and LATS1 (Figure 5B,C). These data strongly suggest that in cardiomyoblasts, intracellular contractility can activate the biochemical axis of the Hippo pathway, which leads to YAP inactivation. This is in stark contrast to the previously described mechanisms in fibroblasts whereby contractility promotes nuclear entry of YAP, independent of biochemical regulation.^[^ ^20^ ^]^

The BCH domain of BNIP‐2 is a highly conserved versatile scaffold that regulates small GTPases such as Rho, Rac, and Ras.^[^ ^23^, ^43^, ^44^ ^]^ To evaluate if BNIP‐2 does impact Rho signaling in cardiomyoblast, we measured the levels of active RhoA (RhoA‐GTP) and phosphorylated myosin light chain in BNIP‐2 knockdown and BNIP‐2 over expressed H9c2 cardiomyoblast. We observed a significant decrease in the active Rho level and phosphorylated myosin light chain in BNIP‐2 knockdown cells (Figure S5A,C, Supporting Information), a direct indication of reduced intracellular contractility in the absence of BNIP‐2. Conversely, overexpression of BNIP‐2 increased levels of active RhoA and phosphorylated myosin light chain (Figure S5B, Supporting Information). Since RhoA is the upstream GTPase that regulates myosin II‐dependent contractility, we further hypothesized that BNIP‐2 might promote cellular contractility through RhoA activation, with the resultant increase in cell contractility being crucial for BNIP‐2 to support YAP inactivation by LATS1 during cardiomyoblast differentiation. To test this hypothesis, we first verified whether force generation by cardiomyocytes is affected by the absence of BNIP‐2. Consistently, BNIP‐2 knockdown significantly reduced the average force generated on microfabricated pillars that mimic in vivo rigidity (Figure 5D,E), suggesting that BNIP‐2 activation of RhoA and myosin light chain activation is required for force generation on the extracellular matrix (ECM). Next, we performed a laser ablation experiment (Figure 5F) to determine the intracellular contractility that should reflect the activation of RhoA by BNIP‐2. Indeed, the knocking down of BNIP‐2 reduced the recoil velocity of actin filaments. Conversely, overexpression of BNIP‐2 increases the recoil velocity by nearly tenfolds (Figure 5G). Hence, BNIP‐2 is required to generate intracellular force and exert traction force on the ECM. With BNIP‐2 knockdown reducing LATS and YAP interaction and that BNIP‐2 activates Rho and cellular contractility, we went on to confirm that BNIP‐2 activation of Rho‐dependent cellular contractility indeed is crucial for YAP inactivation. We compared the levels of YAP phosphorylation at S127 in control cells with those under BNIP‐2 knockdown, rescued with BNIP‐2 overexpression, or rescued with BNIP‐2 overexpression but in the presence of blebbistatin. Our results showed that reduced YAP phosphorylation in BNIP‐2 knockdown cells was restored by BNIP‐2 overexpression. Importantly, blebbistatin treatment effectively blocked the rescue effects by BNIP‐2 overexpression (Figure S5E, Supporting Information). Taken together, RhoA‐mediated YAP inhibition requires cellular contractility in HEK293T, just as in H9c2, further highlighting BNIP‐2/Rho/YAP axis as an important common regulatory mechanism.

At this juncture, we have delineated the multifaceted roles of BNIP‐2 to i) promote cardiomyoblast differentiation through YAP inactivation and cardiac gene expression, and ii) to regulate the intracellular contractility for LATS1 and YAP interaction. Next, we sought to functionally determine whether BNIP‐2 regulation of contractility could directly inactivate YAP by altering YAP intracellular localization. To do so, RhoA activity was pharmacologically modulated using Rho Activator II or RhoA inhibitor C3 toxin (Figure S5C, Supporting Information) and assayed for YAP localization. Consistent with our findings that LATS1 and YAP interaction requires force (Figure 5B,C), and our hypothesis that force regulates YAP intracellular localization in H9c2 cardiomyoblast, a significantly reduced YAP nuclear signal in H9c2 cells was observed upon RhoA activation. In contrast, increased YAP nuclear localization was observed upon the attenuation of cellular contractility by RhoA inhibition (Figure 5H). Finally, we examined how modulating RhoA activity could impact cardiac gene induction. We observed that the treatment with Rho inhibitor reduced the expression of cTnT and Myl2 in ATRA‐treated H9c2 cells (Figure 5I). Strikingly, the introduction of Rho activator could partially rescue the expression of cTnT and Myl2 (Figure 5I). In addition, YAP phosphorylation could also be partially restored by Rho activator in BNIP‐2 knockdown cells (Figure 5I). Collectively, these data show that BNIP‐2 activation of force (i.e., cellular contractility) through RhoA helps scaffold LATS1 and YAP and promotes YAP cytoplasmic localization during differentiation and cardiac gene induction.

**Figure 6:**
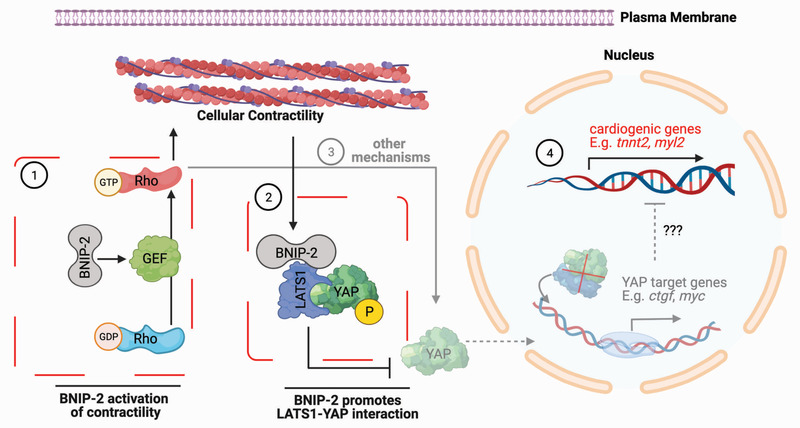
A schematic showing the concerted effects of BNIP‐2 inactivation of YAP for H9c2 cardiomyoblast differentiation. BNIP‐2 is required for both 1) induced cellular contractility and 2) acting as a scaffold for LATS1 and YAP. 1) BNIP‐2 activation of RhoA induces cellular contractility through myosin light chain phosphorylation during cardiac gene induction. 2) Enhanced and/or sustained intracellular contractility is required to promote LATS1‐YAP and LATS1‐BNIP‐2 interaction. BNIP‐2 scaffolds LATS1 and YAP leading to YAP phosphorylation and its failure to enter the nucleus. 3) Other effector mechanisms via RhoA activation that may contribute to the regulation of YAP activities independently of cellular contractility. 4) Together, YAP target genes (e.g., growth genes, *ctgf* and *myc*) are turned off and cardiogenic genes are turned on for cardiomyoblast differentiation. Please refer to main text for more details.

In summary, YAP inactivation through site‐specific phosphorylation and cytoplasmic retention is required for hESC‐CM and H9c2 cardiomyoblast differentiation. Mechanistically, this YAP inactivation process requires the scaffold protein, BNIP‐2, to directly interact with and promote LATS1 and YAP interaction, which is also dependent on the contractility exerted by BNIP‐2‐induced Rho signaling. Importantly, BNIP‐2 plays a multifaceted role in integrating mechanical signaling through RhoA‐induced contractility with the canonical LATS‐YAP pathway to actuate the differentiation process. **Figure** **6** illustrates our proposed mechanism for BNIP‐2 involvement in H9c2 differentiation and the significance is discussed below.

## 3 Discussion

Using a combinatorial approach of biochemical, biophysical, genetic and imaging analyses, we have delineated two distinct but concerted mechanisms of H9c2 cardiomyoblast differentiation mediated by BNIP‐2: i) BNIP‐2 generates cellular contractility by promoting RhoA activity; and ii) BNIP‐2 activation of cellular contractility, in turn, allows BNIP‐2 to promote scaffolding of LATS1 with YAP, leading to YAP inactivation and retention in the cytoplasm, thus switching cardiomyoblast genetic programming from proliferation to sustained cardiac gene expression for differentiation (please refer to the model in Figure 6).

Recent findings that YAP activity can be modulated by Hippo‐dependent and Hippo‐independent events underscore the complexity of the YAP regulatory networks, which adaptively operate under different physiological and pathophysiological processes.^[^ ^18^, ^34^, ^45^, ^46^ ^]^ Accumulating evidence have supported a more nuanced view that biochemical or mechanical regulation of YAP through LATS1 phosphorylation alone may be insufficient to induce YAP activation/inactivation.^[^ ^47^, ^48^, ^49^ ^]^ Rather, multiple biochemical or mechanical pathways seem to exist, which must reach a threshold for YAP activation/inactivation. For instance, despite normal LATS1 activity and YAP S127 phosphorylation, *α*E‐catenin depletion has been reported to result in higher nuclear YAP in hair follicle stem cells, implying that LATS1‐dependent biochemical signal can be bypassed.^[^ ^50^ ^]^ On the other hand, in genetically modified talin1‐null fibroblast that initially lacks nuclear YAP, ECM‐nuclear mechanical coupling was shown to promote YAP nuclear translocation independent of biochemical signaling axes.^[^ ^20^ ^]^ Importantly, while these studies likely represented the extrema in how YAP signaling network can be tuned (i.e., by using mechanobiological perturbation to override biochemical signaling), in many physiological contexts, it is likely that an intricate balancing act between biochemical and mechanobiological cues is at play. This would then implicate the existence of a context‐dependent key molecular regulator that can integrate biochemical (Hippo‐dependent) and mechanical (Hippo‐ independent) inputs to regulate YAP activity through YAP intracellular localization. Our findings in H9c2 cardiomyoblast report a central and multifaceted role of a molecular scaffold, BNIP‐2, that concurrently activates the biochemical and mechanical axes to activate intracellular contractility through RhoA activation and inactivate YAP by directly scaffolding LATS1 to YAP. These findings are highly relevant to in vivo cardiac development as it has previously been shown that nuclear YAP activation in mouse fetal heart not only promotes proliferation but induces the expression of various genes that controls cytoskeletal dynamics.^[^ ^51^ ^]^ In addition, it has been previously suggested that LATS1/2 kinase activity can be activated by GPCR activation through an unknown Rho GTPase activation and altered F‐actin dynamics through a yet elusive mechanism.^[^ ^52^ ^]^ Our findings now show that BNIP‐2 is likely the missing trigger for RhoA activation as well as enhanced LATS1 activity which inactivates YAP during cardiomyoblast differentiation. Furthermore, since proper heart formation requires distinct steps of cardiomyocyte proliferation followed by terminal differentiation,^[^ ^53^ ^]^ our identification of BNIP‐2 as a molecular regulator that integrates mechanical and biochemical regulation of YAP raise an intriguing possibility that BNIP‐2 could function as the in vivo switch between proliferation and terminal differentiation.

The role of scaffold proteins in regulating cell growth, apoptosis, morphogenesis, migration, and differentiation poses an interesting conundrum. As shown in various cellular systems, scaffold proteins enable complex signal processing that may be important for spatiotemporal localization and amplification of signals.^[^ ^36^, ^54^ ^]^ One such example is the scaffolding effects of another BCH domain‐containing protein, BPGAP1, that spatially integrates JNK/ERK signaling. Interestingly, BPGAP1 is autoinhibitory and requires phosphorylation by JNK for its activation to promote MEK partner 1‐ induced ERK activation.^[^ ^36^ ^]^ Hence, there may exist beyond biochemical regulation other forms of regulatory elements to facilitate scaffolding proteins to generate transient, sustained, and even oscillatory signals at the right place and at the right time. Given the prominent roles of mechanical stimuli in influencing key cellular functions (e.g., proliferation and differentiation)^[^ ^20^, ^34^ ^]^ it can be postulated that cell‐ECM or cell‐cell interactions may help provide mechanical cues that influences scaffolding proteins functions. Indeed, previous studies revealed that BNIP‐2 can localize to either cell protrusions or microtubules^[^ ^17^, ^37^ ^]^ and that YAP can localize to the cytoplasm, the nucleus or the adherens junctions.^[^ ^55^ ^]^ Our study revealed that BNIP‐2 colocalizes with LATS1 and the cellular protrusions during cardiomyoblast differentiation. These observations suggest that after the establishment of clear cardiomyocyte‐specific sarcomeric and microtubular structures, BNIP‐2 could be relocalized from the initial cellular protrusions to the microtubule.

Intriguingly, an increased BNIP‐2 expression correlated with the emergence of cardiac gene markers (cTnT and Myl2). Could BNIP‐2 be expressed prior to cardiac genes? Or are they concurrently expressed under similar transcriptional regulations? We postulate the latter based on the transcription factor binding prediction for the presence of promoter sequences upstream of the first exon of BNIP‐2, which responds to the transcription factor FOXP3.^[^ ^56^ ^]^ Interestingly, various studies have shown that ATRA (which is also required for cardiac development) induces the expression of FOXP3.^[^ ^57^, ^58^ ^]^ These observations implicate that more organ systems (e.g., immune, eye, hindbrain, spinal cord, lung, pancreas, and skeleton) that require ATRA during development may be regulated by BNIP‐2. Some of these possibilities are now being investigated.

Work is currently underway to elucidate how RhoA is activated in situ by BNIP‐2 and how force/contractility can regulate the BNIP‐2/LATS1/YAP complex. The activation of GEFs and/or inactivation of RhoGAP through its BCH domain is one possible mechanism. We have recently shown that BNIP‐2 in epithelial cells activate RhoA by scaffolding RhoGEF GEH‐H1 upon microtubule depolymerization^[^ ^28^ ^]^ whereas its homologous BCH domain in p50RhoGAP can control how its adjacent RhoGAP domain functions toward Rho.^[^ ^27^ ^]^ It is currently unknown which RhoGEF or RhoGAP may be involved in the process of BNIP‐2‐dependent cardiomyoblast differentiation. Moreover, it remains an interesting prospect to dissect how cells can sense and generate the contractile force through BNIP‐2 and RhoA, and then transduce such mechanical signals into promoting its biochemical scaffolding function that enables LATS to phosphorylate YAP in the cytoplasm. It is likely that such mechanical force‐dependent process may activate directly or indirectly biochemical activities in one (or more) of the constituents of the BNIP‐2/Rho/Hippo/YAP complex. One interesting possibility could be that LATS1, which was found to bind to actin and favors actin depolymerization^[^ ^59^ ^]^ could have “disengaged” from RhoA‐induced F‐actin structures. Such disengagement could have led to the colocalization of BNIP‐2 and LATS1 at cell protrusions observed in our study, paving the way toward our better understanding of mechanical force in regulating the spatiotemporal modes of biochemical signaling and functions.

On the other hand, previous studies have observed RhoA regulation of YAP activation instead of its inactivation and that effect appears to be less dependent on force.^[^ ^60^, ^61^, ^62^, ^63^ ^]^ For instance, various human disease models (e.g., diffuse gastric cancer, glioblastoma tumorigenicity, abnormal human trabecular meshwork) implicate the activation of YAP that is dependent on RhoA activation. Most of these systems focus on the proliferative effects induced by YAP, which is aligned with the known functions of YAP. Our study, therefore, offers fresh insights into the coupling of force and biochemical interactions during the induction of cardiac genes. To this end, we are investigating how BNIP‐2 could regulate focal adhesion complex, which is also known to be regulated by RhoA^[^ ^61^ ^]^ and linked to YAP activities.^[^ ^61^, ^64^ ^]^

Finally, understanding how the switch from growth to differentiation regimes could be achieved through BNIP‐2‐mediated regulation of the Rho and Hippo‐YAP signaling pathways should help advance further research into potential targets to improve and restore lost functions of heart cells and tissues, and to prevent their malfunctions from developing into heart diseases. Furthermore, since H9c2 cells also show high similarities to primary neonate cardiomyocytes (e.g., hypertrophic responses, hypoxia‐reoxygenation injury, response to RA‐induced differentiation),^[^ ^9^, ^10^, ^11^, ^12^ ^]^ more detailed mechanistic understanding of key signaling nodes at the molecular levels can help translate our findings for future in vivo studies and translational applications.

## 4 Experimental Section

### Cell Culture and Transfection

All cells were grown at 37 °C with 5% CO_2._ H9c2 cells (ATCC) were cultured in Dulbecco's modified Eagle's media (DMEM, Hyclone) supplemented with 10% fetal bovine serum (FBS) (Gibco), 1 × 10^−3^ m sodium pyruvate (Gibco), 100 units mL^−1^ penicillin and 100 mg mL^−1^ streptomycin (Hyclone). Differentiation media contains 1% FBS (Gibco) supplemented with 1 × 10^−6^ m ATRA (Sigma Aldrich). HEK293T cells were cultured in RPMI 1640 medium (Hyclone) supplemented with 10% FBS, 10 × 10^−3^ m HEPES, 100 units mL^−1^ penicillin and 100 mg mL^−1^ streptomycin (Hyclone). Cells were transfected using Polyplus Jetprime according to manufacturer's instructions. Briefly, plasmid and siRNA were mixed with Jetprime reagent in 2:1 or 1:1 ratio, respectively. The complex was allowed to rest at room temperature for 20 min before adding to culture medium. All cells were tested negative for mycoplasma.

### Plasmids and siRNA

For overexpression studies, pXJ40 vector (gift from Dr. Ed Manser, Institute for Molecular and Cell Biology, Singapore) encoding full length human BNIP‐2,^[^ ^24^ ^]^ Lats1, and YAP cDNA were tagged with GFP, mCherry, Flag or HA. pBabe puro eGFP‐YAP1 was a generous gift from Dr. Marius Sudol. pEGFP C3‐Lats1 was a gift from Marius Sudol (Addgene plasmid #19053; http://n2t.net/addgene:19053; RRID:Addgene_19053). GST‐ Rhotekin was kindly provided by Dr. Simone Schoenwaelder, Monash University, Australia). The Paxillin‐mApple plasmid was from Davidson collection (Kanchanawong lab, MBI).

Two siRNA sequences targeting BNIP‐2 were purchased from Dharmacon. Both sequences (5' CGU UAG AAG UUA AUG GAA AUU 3' and 5' GGA UGA AGG UGG AGA AGU UUU 3') were verified by western blotting and RT‐PCR for RNA interference efficiency.

### Antibodies and Chemicals

BNIP‐2 (HPA026843) antibody was purchased from Sigma‐Aldrich. RhoA (sc‐418), Actin (sc‐4778), tubulin (sc‐5286), GAPDH (47724), cTnT (sc‐20025), and Myl2 (sc‐517414) antibodies were purchased from Santa Cruz. MLC2 (#3672), Phospho‐MLC2 Thr18/Ser19) (#3674), MST‐1 (#3682), Phospho‐MST1 (Thr183)/MST2 (Thr180) (#49332), YAP/TAZ (#8418), phospho‐YAP (Ser127) Antibody #4911, Phospho‐TAZ (Ser89) (#59971), Lats1 (#9153), and Phospho‐LATS1 (Ser909) (#9157) were purchased from Cell Signalling Technology. Rabbit IgG (sc‐2027) and mouse IgG (sc‐2025) were from Santa Cruz Biotechnology. HRP secondary antibodies polyclonal antibody against FLAG and polyclonal antibody against HA were from Sigma. All Alexa Fluor dyes and Alexa Fluor Phalloidin were from Life Technologies. For Rho activity assay, Rho inhibitor I (Cat. No. CT04) and Rho activator II (Cat. No. CN03) were from Cytoskeleton, Inc. Dimethyl sulfoxide (DMSO), blebbistatin, and all‐*trans* retinoic acid were from Sigma‐Aldrich.

### hESC CRISPR‐Targeted Knockdown

sgRNAs were designed by identifying NGG PAM sites targeting exon 1, 3, and 4 of the BNIP2 gene locus. Optimized guides were selected using a web browser for selection of sgRNA candidates (http://crispor.tefor.net/) as previously described^[^ ^65^ ^]^ and using the “Rule set 2” scoring model in prioritizing top sgRNA candidates.^[^ ^66^ ^]^ Annealed sgRNA constructs at a final concentration of 0.4 × 10^−6^ m were cloned into 500 ng of *Esp3I*‐digested LentiCRISPRv2 backbone (Addgene #52961) using T4 DNA ligase (catalogue no. M0202, New England Biolabs, NEB). Vectors were transformed via heat shock into OneShot Stbl3 Chemically Competent *E. coli* (catalogue no. C737303, Invitrogen) according to manufacturer's instructions. Plasmid DNA was extracted using Monarch Plasmid Miniprep Kit (NEB) and constructs were confirmed by Sanger sequencing. Each cloned plasmid construct was packaged into lentivirus as described earlier. Prior to transduction, H1‐hESCs were pretreated with 8 µg mL^−1^ polybrene for increased transduction efficiency. Two independent pairs of guides targeting BNIP2 along with a corresponding pair of nontargeting control guides were cotransduced at high multiplicity of infection (MOI) into each well in a 12‐well plate format. hES colonies were selected with 1 µg mL^−1^ Puromycin (Sigma catalogue no. P9620) for 2 d, and subcultured for at least 5 d before differentiation into cardiomyocyte lineage.

### Lentivirus Production

To produce virus containing individual sgRNA constructs, HEK293T cells were cultured in DMEM+10% FBS until 70% confluency before transfection. 10 µg of sgRNA plasmid construct, 7.5 µg of pMDLg/pRRE, 2.5 µg of pRSV‐Rev, and 2.5 µg pMD2.G (Addgene #12251, #12253, and #12259), were cotransfected on a 10 cm dish with 50 µL of PEI and 3 mL of Opti‐MEM I Reduced Serum Medium (catalogue no. 31985070, ThermoFisher Scientific). Following overnight incubation, medium was changed to 5% FBS culture. Supernatant were collected twice after 24 and 48 h, respectively. Pooled supernatant were filtered through PES 0.45 × 10^−6^ m filter and viral particles were concentrated using Lenti‐Pac Lentivirus Concentration Solution (catalogue no. LPR‐LCS‐01, GeneCopoeia) according to manufacturer's instructions. For production of lentivirus particles for generation of stable BNIP‐2 overexpressing H9c2 cells, BNIP‐2 construct was cloned into the pCDH‐CMV‐MCS vector which contains puromycin selection marker. Viral particles were produced in HEK293T cells by cotransfection of pMDLG, pMD2G, PRSV, and pCDH‐CMV‐BNIP‐2 using Viafect (Promega). The supernatant was filtered and harvested after 48 h and used to transduce H9c2 cells.

### Human Embryonic Stem Cell‐Derived Cardiomyocyte (hES‐CM) Differentiation

Culture vessels were coated with Geltrex (catalogue no. A1413202, ThermoFisher Scientific), for at least 30 min prior to seeding. H1 Human embryonic stem‐cell (H1‐hESC) line was maintained in mTeSR1 medium (STEMCELL Technologies) and cultured until 80% confluency prior to seeding for differentiation or subculturing. This study adopted the cardiomyocyte differentiation GiWi (GSK3 inhibitor and Wnt inhibitor) protocol with RPMI differentiation medium as previously described.^[^ ^67^ ^]^ Briefly, 2 d before differentiation, H1‐hESCs were dissociated into single cells with Accutase (Invitrogen) in 37 °C for 5 min and seeded 800 000 cells onto Geltrex‐coated plates in a 12‐well format (200 000 cell cm^−2^) with 10 × 10^−6^ m ROCK inhibitor Y27632 (STEMCELL Technologies). Medium was then refreshed without ROCK inhibitor the following day. At Day 0, cells should achieve about 80% confluency, and were treated with 7 × 10^−6^ m CHIR99021 (catalogue no. 72054, STEMCELL Technologies) in RPMI/B27 without insulin (Gibco). At Day 1, Fresh RPMI/B27 media without insulin was replaced after 24 h. At Day 3, 5 × 10^−6^ m IWP2 (catalogue no. I0536, Sigma Aldrich) was added into conditioned media and fresh media (RPMI/B27 without insulin) at 1:1 ratio. At Day 5, fresh media (RPMI/B27 without insulin) were added without the Wnt inhibitor. Cells were then maintained in RPMI/B27 with insulin (catalogue no. 17504044, Gibco) from day 7 onward and refreshed every 3 d.

### Pharmacological Treatments

For ATRA‐induced differentiation, culture medium was replaced with differentiation medium and supplemented with 1 × 10^−6^ m ATRA. Differentiation medium with fresh 1 × 10^−6^ m ATRA was replaced daily up to day 3. For blebbistatin treatment, differentiating medium containing 1 × 10^−6^ m ATRA was supplemented with 20 × 10^−6^ m blebbistatin. For Rho activator and inhibitor assay, cells were treated with 1 µg mL^−1^ of activator or inhibitor for 24 h before fixing the cells with 4% warm PFA.

### RT‐PCR Experiment

Total RNA was harvested using RNeasy Mini Kit (Qiagen) and cDNA was generated using SuperScript IV VILO MasterMix (ThermoFisher Scientific) according to the manufacturer's protocol. For RT‐PCR, equal concentration of cDNA was analyzed using SsoFast EvaGreen Supermix (BioRad) according to the manufacturer's protocol. A BioRad CFX96 Touch Real Time PCR machine was used to detect cDNA levels of respective genes across samples. Sequences of specific primers used for RT‐PCR in this paper are summarized in Table S1 (Supporting Information).

### Immunoprecipitation and Western Blot

Endogenous immunoprecipitation was performed using PureProteome Protein A/G Magnetic Bead System (LSKMAGA10) according to the manufacturer's protocol with slight modification. Briefly, cells were lysed in ice cold RIPA ((50 × 10^−3^ m Tris (pH 7.3), 150 × 10^−3^ m sodium chloride, 0.25 × 10^−3^ m EDTA, 1% Triton X‐100, 1% w/v sodium deoxycholate, and a mixture of protease inhibitors) and split equally to be precleared with IgG control or BNIP‐2 antibody for 45 min at 4 °C. Protein A magnetic beads were then added and the sample was further incubated for 2 h with constant shaking at 4 °C. Samples were washed thrice with ice cold 1× PBS before adding loading dye and denatured at 95 °C for 10 min.

For exogenous co‐immunoprecipitation, anti‐Flag‐tagged or anti‐HA‐tagged magnetic beads from Sigma‐Aldrich were incubated with cell lysates lysed in ice cold RIPA for 1 h at 4 °C prior to washing thrice with 1X PBS. Immunoprecipitation for the active (GTP‐bound) form of RhoA was performed as described previously.^[^ ^44^ ^]^ Western blot was performed as previously described.^[^ ^22^ ^]^ Blots were analyzed using Bio‐Rad clarity ECL substrate (1:3000 dilution) in a ChemiDoc Touch (Bio‐Rad) developer. For endogenous IP, Clean‐Blot IP Detection Reagent (HRP) (#21230) from ThermoFisher Scientific was used at 1:500 dilution.

### LATS1 In Vitro Kinase Assay

The in vitro kinase assay was performed as previously described^[^ ^68^ ^]^ with slight modification. BNIP‐2 protein was in vitro translated using 250 ng of Pxj40‐HA‐BNIP‐2 plasmid and the PURExpress In Vitro Protein Synthesis Kit (NEB # E6800S) following the manufacturer's protocol. LATS1 and YAP were endogenously immunoprecipitated, respectively, using PureProteome Protein A/G Magnetic Bead System (LSKMAGA10). 80% confluent HEK293T cells were lysed with the mild lysis buffer, M‐PER Mammalian Protein Extraction Reagent (Thermo Fisher #78501) supplemented with protease inhibitor, sodium orthovanadate and beta glycerophosphate. Respective cell lysates were incubated with LATS1(CST #3477S) and YAP (CST #4912) antibodies overnight in a cold room. PureProteome Protein A/G Magnetic Bead System (LSKMAGA10) was added to the cell lysates and incubated for an hour in the cold room. 0.1 m Glycine pH 2.0 and pH 8.0 were added sequentially to elute the beads‐bound YAP protein. The kinase assay was performed at 30 °C for 30 min, by mixing the beads‐bound LATS1, eluted YAP and in vitro translated BNIP‐2 with 1× kinase buffer (CST #9802) and 500 × 10^−6^ m cold ATP (NEB # P0756S). 4× SDS sample buffer was added to stop the reaction, and samples were heated at 85 °C for 10 min.

### Immunofluorescence Staining

For fluorescence staining of cells, cells were fixed with warm (37 °C) 4% paraformaldehyde (PFA) at 37 °C for 15 min. For super‐resolution imaging, an additional quenching step using freshly prepared 0.01% sodium borohydride was used to quench the fixation. After fixation, cells were washed thrice with 1× PBS and left on a shaker for a further 5 min at room temperature. Cells were permeabilized with blocking buffer (3% bovine serum albumin, 0.1% Triton X‐100 in 1× PBS) for 30 min at room temperature. After permeabilizing with blocking buffer, cells were incubated with respective primary antibodies for 45 min at room temperature. Thereafter, cells were washed thrice with 1× PBS. For secondary antibody staining cells were incubated in Alexa Fluor conjugated secondary antibodies for 45 min in dark. Finally, cells were washed thrice with 1× PBS. Staining of actin with Alexa Fluor conjugated phalloidin was performed directly after cell fixation with 4% PFA.

### Luciferase Assay

The luciferase assay to quantify YAP activity was performed by transfecting cells with the 8xGTIIC‐luciferase plasmid and using renilla luciferase plasmid (PRL‐TK) as the internal control. Cells transfected only with renilla plasmid were used as a negative control. Briefly, cells were seeded to 80% confluency and transfected with luciferase and renilla plasmid in a 10:1 ratio to assay for YAP activity and prevent over saturation of renilla signal. The Promega Dual‐Luciferase Reporter Assay System (#E1910) was used to quantify luciferase and renilla signal following the manufacturer's protocol. The Promega Glomax Discover plate reader was used to read and record the luciferase signal.

### Confocal and Structured Illumination Microscopy

Confocal imaging was performed using Yokogawa CSU‐W1 Spinning Disk microscope (Nikon) equipped with a 60× (NA 1.20) water immersion lens or 100× (NA 1.45) oil immersion objective lens.^[^ ^69^ ^]^ Super‐resolution imaging was performed using Nikon Structured Illumination Microscope (N‐SIM) equipped with a 100× oil immersion lens taking 15 images (3 directions × 5 phases) for one frame of reconstructed images. SIM reconstruction was performed using NIS‐Elements AR.

### Pillar Array

PDMS micropillars were fabricated as described earlier.^[^ ^70^ ^]^ The dimensions of pillars were: diameter = 2.1 µm with height = 4.1 µm, spring constant *k* = 55 nN µm^−1^. Briefly, cells were seeded on fabricated micropillars overnight and stained with CellTracker Green CMFDA Dye (ThermoFisher Scientific) for 30 min at 37 °C. Single cells and the pillars underneath the cells were imaged using Olympus IX‐81 inverted microscope, UPLSAPO 60XW/1.2NA water immersion objective. Quantification was performed as described earlier.^[^ ^70^ ^]^

### Laser Ablation

Thirty minutes prior to imaging, H9c2 cells were stained with CellMask Actin Tracking Stain (ThermoFisher Scientific, Cat# A57243). To measure recoil velocity due to tensional release of actin filaments within the cells, distinctive features were monitored before, during and after UV laser ablation. Spatiotemporal information of the distinctive features after UV ablation was obtained and quantified using MTrackJ plugin in Fiji imageJ. Fitting of calculated distance between points of distinctive features was carried out using a linear function and a single exponential function. The single exponential function was used for datasets with slow recoil velocity to obtain slow elastic response of the actin network mesh upon ablation. Next, recoil velocity was calculated as the derivative of the above mentioned functions using methods mentioned in earlier literatures.^[^ ^71^, ^72^ ^]^

The linear function is

(1) $$f\;\left(t\right)=p_{1}\;t+p_{2}$$

where *t* is the approximate time when UV laser shutter is opened, *p*_1_ is the deformation speed for linear model, and *p*_2_ is the initial length between points of distinctive features at *t* = 0 s for linear model.

The single exponential model is

(2) $$f\;\left(t\right)=\;a+Ae^{\left(\frac{-1\left(t-b\right)}{\;\tau }\right)}$$

where *t* is the approximate time when UV laser shutter is opened, *a* is the initial length between points of distinctive features at *t* = 0 *s* for exponential model, *τ* is the ratio of Young's modulus to viscosity, and *A* and *b* are arbitrary constants.

The double exponential function was used for datasets with fast recoil velocity to obtain fast elastic response of the actin network mesh upon ablation.

The double exponential model is

(3) $$f\;\left(t\right)=\;a+A_{1}e^{\left(\frac{-1\left(t-b\right)}{\;\tau_{1}}\right)}+A_{2}e^{\left(\frac{-1\left(t-b\right)}{\;\tau_{2}}\right)}$$

where *t* is the approximate time when UV laser shutter is opened, *a* is the initial length between points of distinctive features at *t* = 0 *s* for exponential model, *τ*_1_ and *τ*_2_ are the ratios of Young's modulus to viscosity, and *A*_1_, *A*_2_, and *b* are arbitrary constants.

### Bio‐ID Proximity Labeling

Human BNIP‐2 was PCR subcloned into the NheI site, in between the 3HA tag and TurboID, of 3XHA‐TurboID‐NLS_pCDNA3 (A kind gift from Alice Ting—Addgene plasmid # 107171) using the following primers (Fwd: CCGCTAGC GAAGGTGTGGAACTTAAAGAAGAATG and Rev: CCGCTAGC CTGTTCATTTTTCGGTTCATCTTG). The NLS of the 3XHA‐TurboID‐NLS_pCDNA3 and 3XHA‐BNIP‐2‐TurboID‐NLS_pCDNA3 were removed by site‐directed mutagenesis PCR using primer pair (CGGTCTGCCGAAAAG TG ATTCAGCAGGGCCGAC and GTCGGCCCTGCTGAATCACTTTTCGGCAGACCG). HEK293T transfected with respective plasmid were incubated in media containing 0.5 × 10^−6^ m Biotin (Sigma) 24 h after transfection. Cells were harvested in RIPA buffer after 18 h. Enrichment of biotin‐labeled proteins was achieved using streptavidin magnetic beads (Pierce, ThermoFisher Scientific), and subjected to SDS‐PAGE (Biorad). Gel stabs were processed for mass spectrometry (MS) analysis on a TripleTOF 5600 system (AB SCIEX) in Information Dependent Mode. MS spectra were acquired across the mass range of 350–1250 m/z in high‐resolution mode (>30 000) using 250 ms accumulation time per spectrum. ProteinPilot 5.0 software Revision 4769 (AB SCIEX), capable of using the Paragon database search algorithm (5.0.0.0.4767) and the integrated false discovery rate (FDR) analysis function, was used for peptide identification and quantification against a database consisting of 20190919_SwissProt_human_20659_CRAP_Gel.fasta (total 20659 entries). Only proteins with ≤1% global FDR and distinct peptides with ≤5% local FDR were used for further analysis. Peptides identified with confidence interval ≥95% were considered. The interactome of BNIP‐2 was determined after subtraction of the background provided by the 3XHA‐TurboID_pCDNA3.

### RNA Sequencing

Total RNA was harvested from samples using RNeasy Mini Kit (Qiagen) according to manufacturer's protocol. Total RNA was sent to BGI Genomics Co., Ltd. for RNA sequencing in Shenzhen, China. Samples were sequenced using BGISEQ‐500 paired‐end platform, generating about 26.18 M reads per sample on average. The average mapping ratio with reference genome is 95.70%, the average mapping ratio with gene is 84.11%; A total of 21 877 genes were detected. The sequencing reads which contained low‐quality, adaptor‐polluted and high content of unknown base (N) reads, were removed before downstream analyses.

### Image Analysis

All image processing was performed using either ImageJ software or MATLAB custom‐written script. For focal adhesion size and number quantification, cell edges were identified by analyzing the DIC images, each focal adhesion was identified by analyzing the RFP channel images (focal adhesion staining) via adjusting threshold according to the current images, and by applying ImageJ analysis “Analyse Particles.” The number of focal adhesions per cell was calculated from the number of ROI identified from “Analyse Particles.” For YAP nuclear versus cytoplasmic ratio quantification, signal intensity in the nucleus and a region just outside the nucleus was quantified. For cell alignment quantification, ImageJ plugin “Directionality” was used to calculate dispersion, and a MATLAB custom script was used to generate the rose plot.

### Statistical Analysis

Data comparisons throughout the manuscript are presented as mean ± standard error of the mean (SEM) and plotted using GraphPad Prism 8. All data sets were analyzed using two‐tailed Student's *t*‐test or analysis of variance (ANOVA) with GraphPad Prism 8. In cases whereby, samples do not meet normality criteria, a nonparametric test was used. A *p*‐value of less than 0.05 was considered statistically significant (* indicates *p* < 0.05, ** indicates *p* < 0.01, *** indicates *p* < 0.001, and **** indicates *p* < 0.0001). All experiments involving statistical comparison were performed at least three times.

## Conflict of Interest

The authors declare no conflict of interest.

## Author Contributions

Experiments were conceived and designed by D.C.P.W., J.X., M.A.J., P.K., and B.C.L. Experiments were performed by D.C.P.W., J.X., M.P., J.W.A., M.L.C.J., and T.T. Bio‐ID proximity labeling was performed by T.W.C. and N.J.W.L. D.C.P.W., J.L.D., P.K., and B.C.L. wrote the manuscript. All authors commented on the manuscript and contributed to it.

## Supporting information

Supporting Information

## Data Availability

The data that support the findings of this study are available on request from the corresponding author. The data are not publicly available due to privacy or ethical restrictions.
