# Supplementary material for: BNIP‐2 Activation of Cellular Contractility Inactivates YAP for H9c2 Cardiomyoblast Differentiation
Source: Adv Sci (Weinh). 2022 Aug 17;9(31):2202834. doi: 10.1002/advs.202202834 (PMC9631078; doi:10.1002/advs.202202834)
Supplement: Supplementary file 1 — Supporting Information [file ADVS-9-2202834-s001.pdf]

ADVANCED SCIENCE
Open Access

Supporting Information

for *Adv. Sci.*, DOI 10.1002/advs.202202834

BNIP-2 Activation of Cellular Contractility Inactivates YAP for H9c2 Cardiomyoblast Differentiation

Darren Chen Pei Wong, Jingwei Xiao, Ti Weng Chew, Meng Pan, Chang Jie Mick Lee, Jing Wen Ang, Ivan Yow, T. Thivakar, Matthew Ackers-Johnson, Nicole Jia Wen Lee, Roger Sik-Yin Foo, Pakorn Kanchanawong* and Boon Chuan Low**

Supporting Information

BNIP-2 activation of cellular contractility inactivates YAP for H9c2 cardiomyoblast differentiation

Figure Legends

Supplementary Figure 1. BNIP-2 is highly expressed in the heart.

A

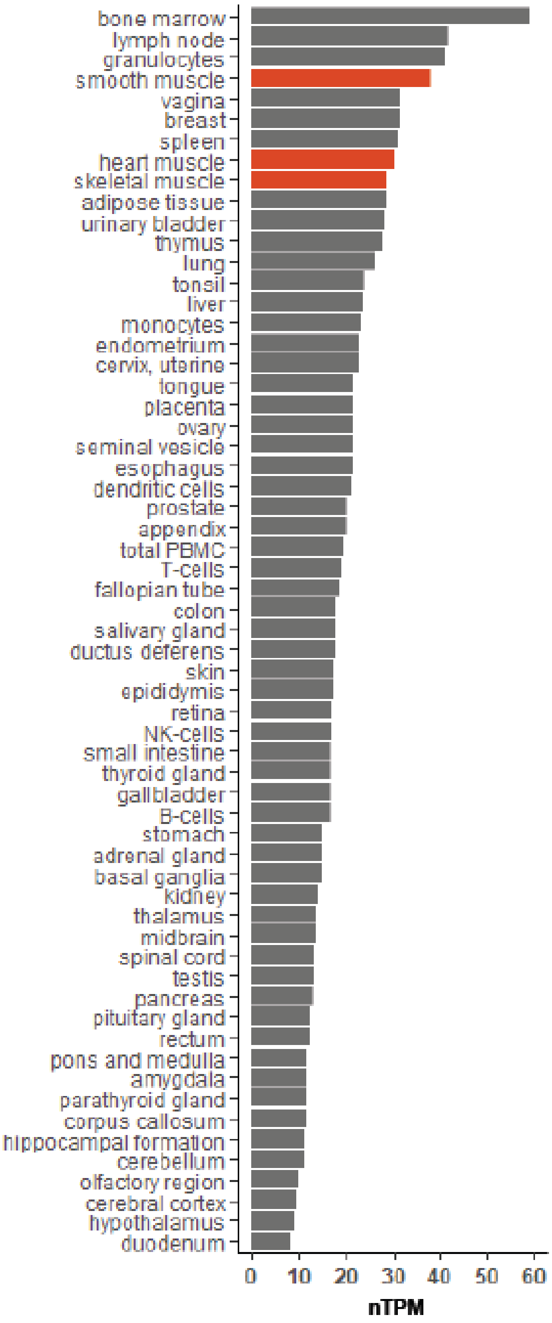

B

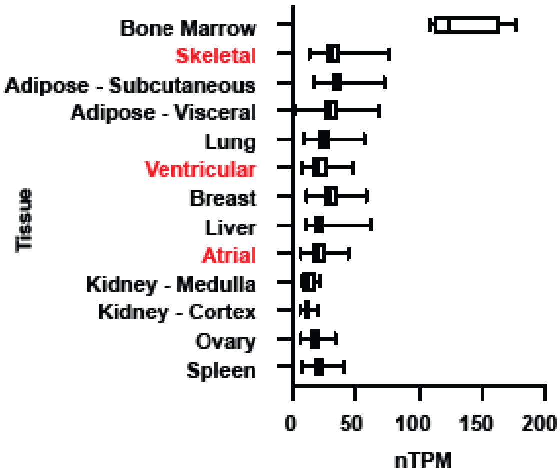

Tissue	n
Bone Marrow	4
Skeletal	803
Adipose-Subcutaneous	663
Adipose-Visceral	541
Lung	578
Ventricular	432
Breast	459
Liver	226
Atrial	429
Kidney-Medulla	4
Kidney-Cortex	85
Ovary	180
Spleen	241

C

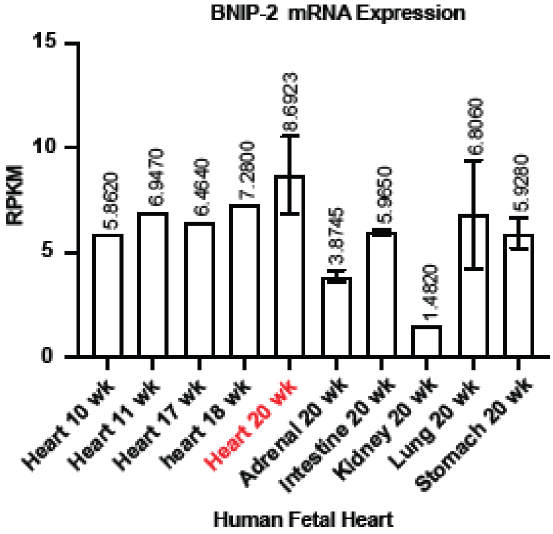

Supplementary Figure 1. BNIP-2 is highly expressed in the heart. A, The consensus dataset obtained from combining HPA, GTEx and FANTOM5 transcriptomics datasets show relatively higher BNIP-2 TPM (transcript per million) in heart and muscle tissues. Data obtained from the Human Protein Atlas and is available from http://www.proteinatlas.org (refer to main text for specific citations). B, Several organs of mesoderm lineage show relatively higher BNIP-2 RNA expression. Data obtained from Human Protein Atlas and is available from http://www.proteinatlas.org. n values for each organ is tabulated in the table below (refer to main text for specific citations). C, BNIP-2 Reads Per Kilobase of transcript per Million mapped reads (RPKM) increases during human fetal heart development. BNIP-2 expression is higher compared to other tissues. Bars represent mean ± S.E.M, n=1-3 replicates per tissue. Available from: Gene [BNIP-2]. Bethesda (MD): National Library of Medicine (US), National Center for Biotechnology Information; 2004 – [Cited 2020 June 01][30]. Available from: https://www.ncbi.nlm.nih.gov/gene/.

Supplementary Figure 2. BNIP-2 induced cardiac gene expression was indpendent of p38MAPK.

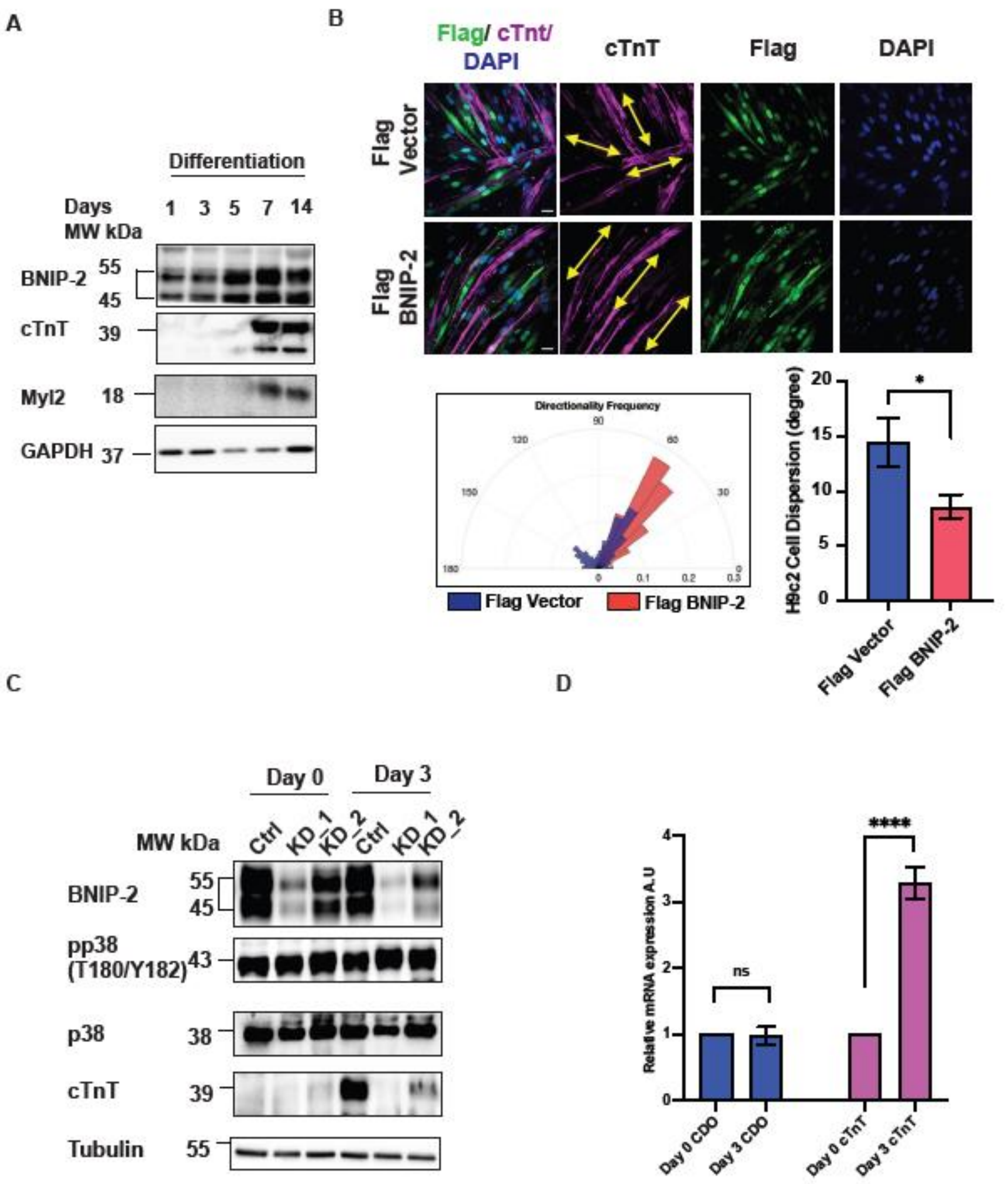

Supplementary Figure 2. BNIP-2 regulates H9c2 cardiomyoblast differentiation independent and p38MAPK. A, hESC cells were differentiated for 14 days and harvested at days 1, 3, 5, 7 and 14 for immunoblot analysis. BNIP-2 expression increased in hESC derived cardiomoyocytes. A higher BNIP-2 expression corelated

with higher cTnT and Myl2 expression, and preceded the expression of cTnT and Myl2 at Day 5, n=3. B, BNIP-2 promoted the alignment of H9c2 cardiomyoblast after 3 days of ATRA treatment. Merged: combined signals from cTnT (magenta), Flag Vector or BNIP-2 (green), and DAPI (blue). Scale bar=30 μm. Rose plot shows a representative directionality frequency and bar graph shows quantification of dispersion angles, mean ± S.E.M, n=10 fields of views. p-value was calculated by the Student's t-test: *$p<0.05$ C, ATRA treatment of H9c2 cells for three days resulted in enhanced cTnT expression in BNIP-2 expressing control cells, but no p38 phosphorylation was observed. The knocking down of BNIP-2 did not affect p38 phosphorylation in both ATRA treated (Day 3) or non-treated control (Day 0) cells, n=3. D, RT-PCR analysis revealed no statistical differences in CDO expression between non-differentiated (Day 0) and cTnT-expressing differentiated cells (Day 3 ATRA treated). The graph represents mean ± S.E.M and unpaired students T-test was used, n= 5. p-value was calculated by the Student's t-test: ** $p<0.01$.

Supplementary Figure 3. YAP is inactivated for H9c2 cardiomyoblast differentiation.

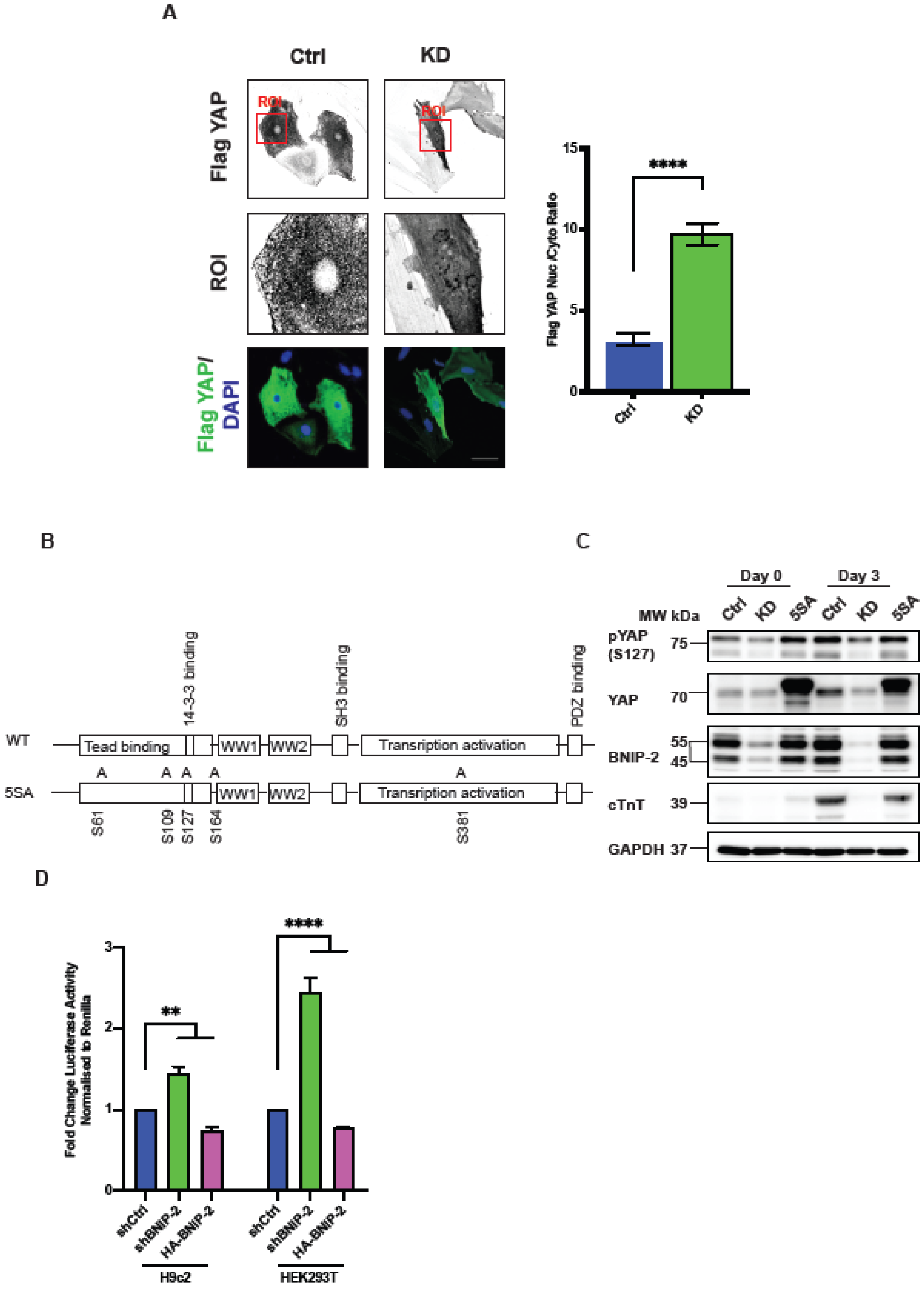

Supplementary Figure 3. YAP is inactivated for H9c2 cardiomyoblast differentiation. A, BNIP-2 knockdown reduced the nuclear localization of overexpressed YAP in H9c2 cardiomyoblast. H9c2 control cells or siRNA knockdown cells were transfected with Flag YAP and stained with Flag antibody. Quantification on the right represents mean ± S.E.M, n=26. p-value was calculated by the Student's t-test: **** $p<0.0001$. B, Schematic of five serine-to-alanine mutations in YAP construct. The 5SA YAP is refractory to LATS1 phosphorylation. C, H9c2 cells were treated with ATRA for three days to induce expression of cardiac marker cTnT. BNIP-2 knockdown and overexpression of YAP (5SA) mutant reduced the expression of cardiac cTnT in H9c2 cardiomyoblast, n= 4. D, Luciferase reaction showing fold change in YAP activity normalised to the internal Renilla control. BNIP-2 knockdown induced significantly higher YAP activity and conversely, BNIP-2 overexpression reduced YAP activity, n=4. p-values were calculated by the Student's t-test: **$p<0.01$ and **** $p<0.0001$.

Supplementary Figure 4. LATS1 is an interacting partner of BNIP-2.

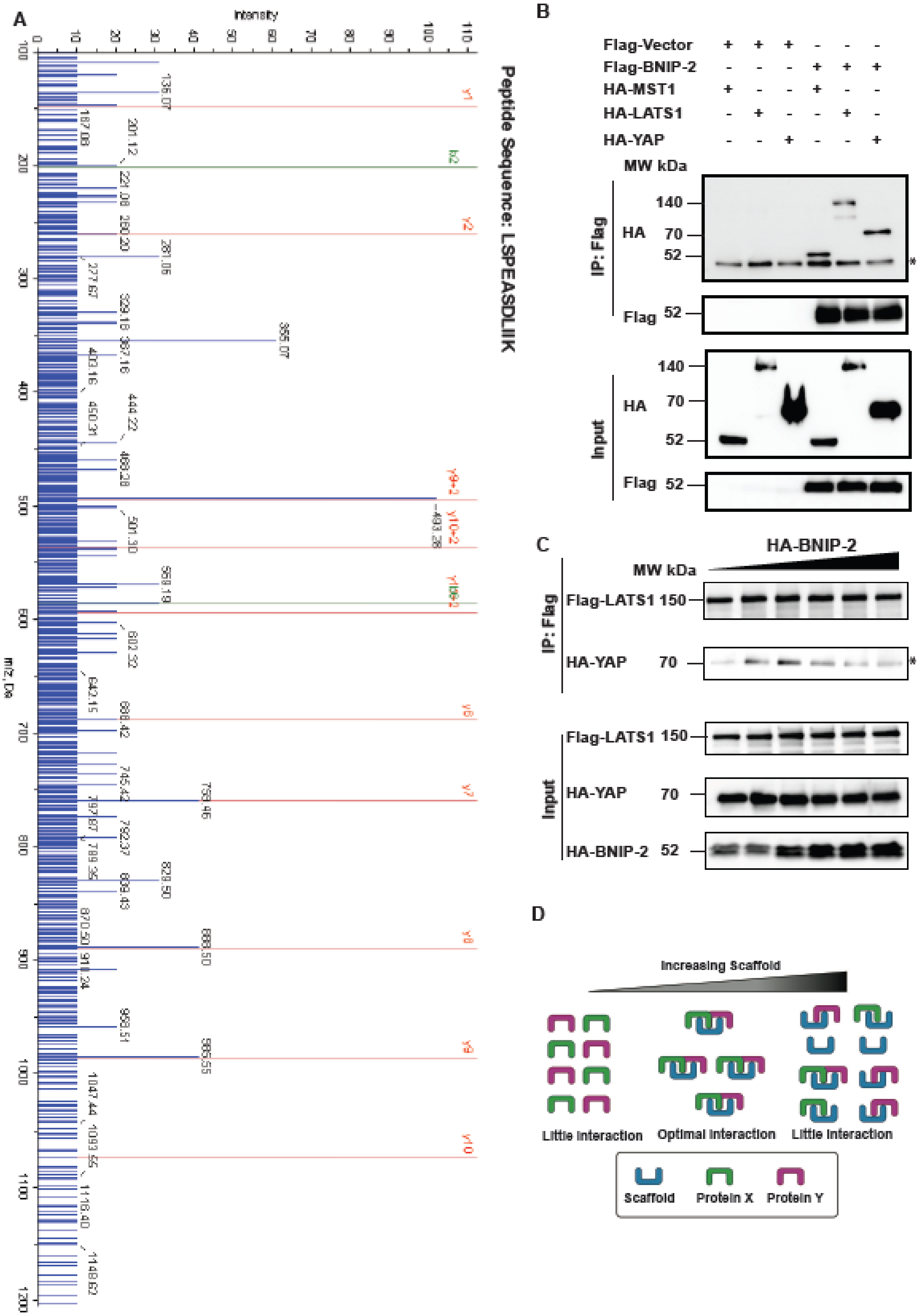

Supplementary Figure 4. LATS1 is an interacting partner of BNIP-2. A, Identification of a peptide motif that maps to LATS1 protein with a confidence interval ≥ 95%. B, Immunoblot showing Flag BNIP-2 pulls down HA-LATS1, MST1 and YAP in HEK293T cells overexpressing the proteins. The * denotes non-specific antibody heavy chain, n=3. C, Immunoblot showing an increase followed by a decrease (blot denoted by *) in HA-YAP and Flag-LATS1 interaction in the presence of increasing BNIP-2 overexpression in HEK239T cells, n=3. D, A schematic showing the optimal concentration of scaffold protein (blue) facilitates an optimal interaction between proteins X and Y. In absence of a scaffold, proteins X and Y are unable to interact. On the contrary, too much scaffold will result in proteins X and Y binding individually to the scaffold.

Supplementary Figure 5. BNIP-2 is required for RhoA activation and myosin phosphorylation.

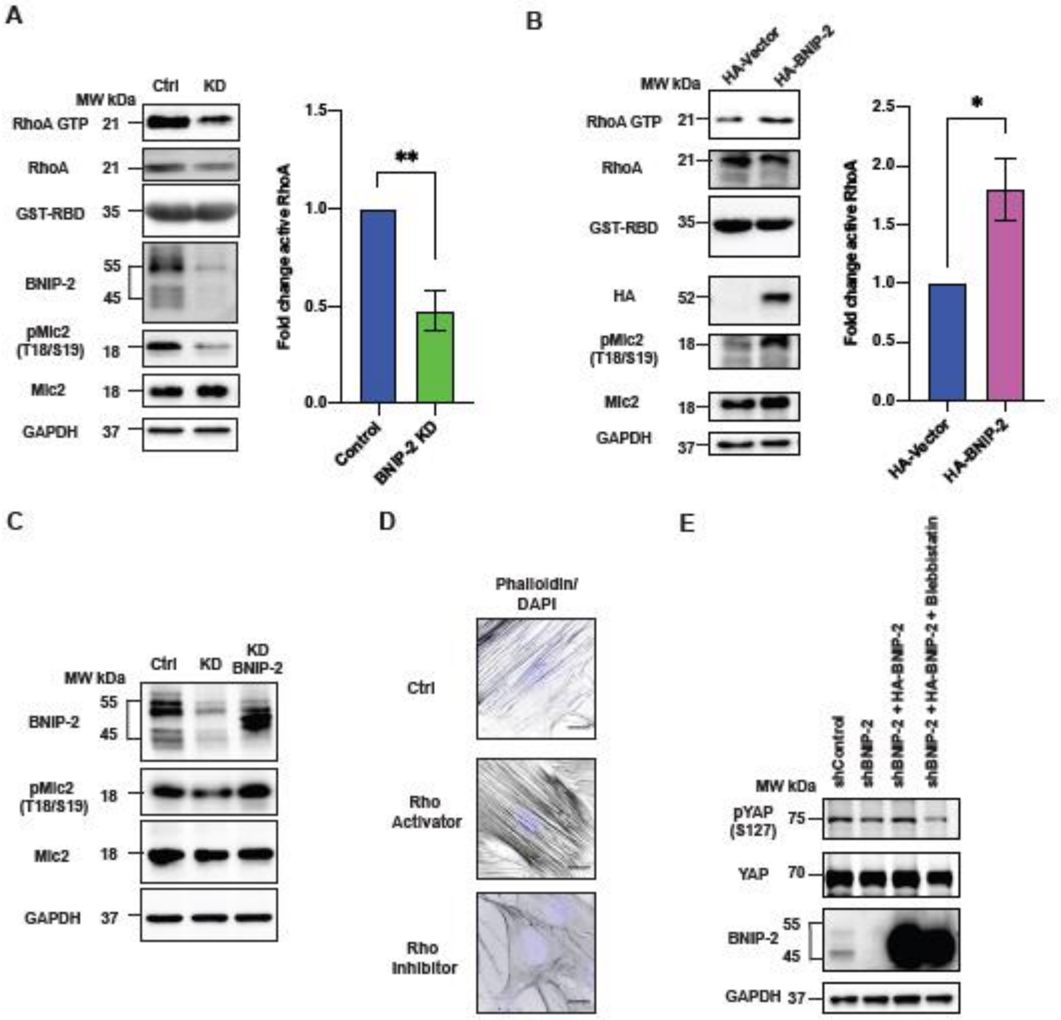

Supplementary Figure 5. BNIP-2 is required for RhoA activation and myosin phosphorylation. A. Immunoblot and quantification showing pulldown of active RhoA-GTP using GST-RBD beads (pulls down GTP-bound form of RhoA) was lesser in BNIP-2 knockdown H9c2 cells, n=3. p-value was calculated by the Student's t-test: **$p<0.01$ B, Immunoblot and quantification showing pull down of active RhoA-GTP using GST-RBD beads was increased with overexpression of HA-BNIP-2, n=3. p-value was calculated by the Student's t-test: *$p<0.05$ C, Immunoblot showing BNIP-2

knockdown reduced active myosin light chain. The re-introduction of BNIP-2 was able to rescue myosin light chain phosphorylation, n=3. D, The H9c2 F-actin network was altered by the addition of Rho activator (1 μM) or inhibitor (1 μM). DAPI (Blue) stains for the nucleus and Phalloidin (grey) stains for F-actin. Addition of Rho activator resulted in intense stress-fibre appearance, while the addition of Rho inhibitor reduced visible stress-fibre. Scale bar = 30 μm. E, Immunoblot showing BNIP-2 knockdown in HEK293T cells reduced levels of pYAP(S127) that was rescued with the addition of HA-BNIP-2. However, addition of blebbistatin (20 μM) reversed the rescue effects of HA-BNIP-2, n=3.

Supplementary Table 1. List of RT-PCR primers used in this study.

Gene	Primer	Sequence (5’-3’)
BNIP-2	Forward	GGT GGA GAA GTT GAC CTG GA
	Reverse	ATG GTC CTG CTC TCC AAT CC
CDO	Forward	AGC ATG CTG GGA AAT ACA CTT
	Reverse	GTC TCG TTC ATC GTT CTG AGA
TnnT2	Forward	GAA GAT TCT GGC AGA GAG GAG
	Reverse	TGA TCC TGT TTC GCA GAA CGT
CTGF	Forward	GAG GAA AAC ATT AAG AAG GGC AAA
	Reverse	CGG CAC AGG TCT TGA TGA
GAPDH	Forward	ATG TTT GTG ATG GGT GTG AA
	Reverse	ATG CCA AAG TTG TCA TGG AT
Myc	Forward	AGT CAG GGT CAT CCC CAT CA
	Reverse	AGC TAC GCT TCA GCT CGT TT
Axl	Forward	CCT GCT CTG GCT TCA AGA TG
	Reverse	CGT GGG ATG TCT GGA AAC C
